# Effect of palliative radiotherapy and cyclin-dependent kinase 4/6 inhibitor on breast cancer cell lines

**DOI:** 10.1007/s00210-025-03878-6

**Published:** 2025-03-04

**Authors:** Marwa Sharaky, Shereen M. El Kiki, Heba Effat, Heba H. Mansour

**Affiliations:** 1 Pharmacology Unit, Cancer Biology Department, National Cancer Institute, Cairo University, Cairo, Egypt; 2 Health Radiation Research Department, National Center for Radiation Research and Technology, Egyptian Atomic Energy Authority, P.O. Box 29, Nasr City, Cairo Egypt; 3 Medical Biochemistry and Molecular Biology Unit, Cancer Biology Department, National Cancer Institute, Cairo University, Cairo, Egypt

**Keywords:** Cyclin-dependent kinase 4/6 inhibitor, Abemaciclib, Irradiation, Breast cancer

## Abstract

The most prevalent disease in the world and the main reason for women mortality from cancer is breast cancer. The recommended treatment for hormone receptor-positive metastatic breast cancer (MBC) is cyclin-dependent kinase 4/6 inhibitor (CDK4/6i), Abemaciclib. Radiotherapy (RT) is one of the main options to control breast cancer. This work intended to examine the impact of CDK 4/6i and palliative radiation on human breast cancer cell lines. Breast cancer cell lines (MCF7, MDA-MD-468, and MDA-MD-231) were treated with varying doses of Abemaciclib and left to incubate for 48 h. Different radiation doses were applied to the lines that had the best IC50. The intrinsic treatment objectives for MBC are presented in this study, along with the PI3K/AKT/mTOR pathway; CDK4, CDK6, and the NF-κβ/TGF-β pathway; BAX/BcL2, P53; caspase-3, caspase-6, caspase-7, caspase-8, and caspase-9; cytokeratin 18 (CK18); cycloxygenase-2 (COX2); IL-6; IL1β; matrix metalloproteinases (MMP2 and MMP9); and oxidative stress markers. The biochemical assays revealed that abemaciclib hindered the progression of breast cancer cells MDA-MB-231 and MCF-7 and enhanced RT (10 Gy) by provoking cell cycle arrest throughout the restraint of CDK4 and CDK6 expression and increasing apoptosis, in addition to decreasing the PI3K/AKT/mTOR and NF-κβ/TGF-β pathway expression; inhibiting CK18 and COX2 activity; boosting the protein concentration of BAX and P53; and decreasing Bcl-2, IL-6, IL-1β, MMP2, and MMP9, modulating oxidative stress markers. These results implied potential effects of radiation and CDK4/6i abemaciclib on breast cancer cell lines.

**Graphical Abstract:**

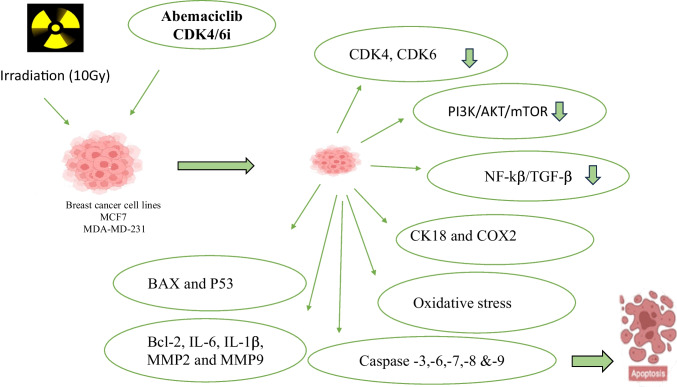

**Supplementary Information:**

The online version contains supplementary material available at 10.1007/s00210-025-03878-6.

## Introduction

Breast cancer is the most common malignancy in women and the primary cause of cancer-related mortality (Jiang and Li 2021; Anwer et al. 2022). Breast cancer is categorized into numerous subgroups based on the countenance of the human epidermal growth factor receptor 2 (HER2), progesterone receptor (PR), or estrogen receptor (ER) (Mahmoud et al. 2023). The second common cause of death for breast cancer patients is metastasis. The bone is the supreme mutual location of breast cancer metastasis, accounting for 70% of all cases of MBC, especially in the ER + subtype. Acquired pharmacological resistance elevates the chance of recurrence (Hellinger et al. 2020; Aggarwal et al. 2021). Recurrence percentages for breast cancer have amended when radiotherapy is combined with other cytotoxic or targeted therapies (Pesch et al. 2021).

It has been shown that a group of cyclin proteins, which activate serine threonine kinase (cyclin-dependent kinases), tightly manages the cell cycle progression in mammals (Corona and Generali 2018; Barkat et al 2020). Following DNA damage, the cell cycle is an essential regulator of cell division, growth, and proliferation. It regulates the switch from the quiescent state (G0 phase) to cell proliferation and passages through checkpoints. All cells activate cyclin-dependent kinases (CDKs) during DNA synthesis (S phase). CDKs must bind to a cyclin subunit to be catalytically active. CDK4/6 proteins arbitrate the passage from the G0/G1 phase to the S phase of the cell cycle (Asghar et al. 2015).

Abundant preclinical findings have revealed that CDK4/6 is essential for breast cancer cell for enduring the ability to cause tumors. Cell cycle progression is synchronized by phosphorylating the tumor suppressor protein retinoblastoma (Rb) through the formation of cyclin-CDK complexes, mostly mediated by CDK4 and CDK6 interaction with D-type cyclins (Niu et al. 2019; Shikanai et al. 2022). The cellular processes in the breast cancer cell were disrupted due to the disorganization of the CDK4/6 pathway (Niu et al. 2019; Piezzo et al. 2020). As a family of tyrosine kinase inhibitors, CDK4/6 inhibitors (CDK4/6i) reduce phosphorylation of Rb protein and preclude cell cycle progression (Shikanai et al. 2022).

The US Food and Drug Administration (FDA) has accepted three CDK4/6i, including palbociclib (PD0332991), ribociclib (LEE011), and abemaciclib (LY2835219), for the remediation of HR + /HER2 − or MBC in combination with endocrine therapy (Bilgin et al. 2017).

The ATP-binding sites of CDK4 and CDK6 were inhibited by abemaciclib (verzenio), a highly selective CDK4/6i. The breast cancer cell lines treated with abemaciclib inhibited phosphorylation of Rb protein in a concentration-dependent manner and prevented cell cycle progression from the G1 to the S phase, which led to apoptosis and senescence (Niu et al. 2019; Johnston et al. 2023).

Moreover, previous studies revealed that abemaciclib mediated antiproliferative effects on both hormone receptor–positive cell line (MCF-7) and the TNBC (MDA‐MB‐231 and MDA‐MB‐468). They demonstrated that abemaciclib treatment resulted in significant apoptotic cell death in MCF-7, MDA‐MB‐231, and MDA‐MB‐468 cells via G0/G1 arrest and phosphorylation of RB1, chromatin condensation, upregulation of caspase‐3 and Bax levels, and downregulation of Bcl‐2 (Anwer et al. 2022; Ozman et al. 2021; Torres-Guzmán et al. 2017).

Radiotherapy (RT) is an efficient treatment option in breast cancer, usually used after surgery to prevent recurrences and metastases (Zhang et al. 2019; Mahmoud et al. 2023; Becherini et al. 2023). Radiation is utilized to demolish cancer cells as it produces ions and rests energy in the cells it pierces. This deposited energy has the ability to either terminate cancer cells or cause genetic changes that terminate cancer cells (Haussmann et al. 2020). Ionizing radiation causes direct DNA strand breaks or produces free radicals, which in turn persuades DNA or other cell components to be destroyed. DNA damage response is triggered by DNA lesions and is necessary to initiate downstream signaling pathways such as apoptosis, cell cycle arrest, and DNA repair. Because breast cancer cells differ in their biology and molecular characteristics, they react differently to radiation (Masoudi-Khoram et al. 2020). Radiation preferentially kills rapidly proliferating cancer cells due to their poor DNA repair mechanisms. The G2 and M phases are the most radiosensitive phases of the cell cycle (Huang and Zhou 2020).

A subdivision of patients treated in the PALOMA trials was prospectively assessed in a clinical environment when palliative RT and CDK4/6 inhibitors were combined (Becherini et al. 2023). Data considering the safety and effectiveness of RT with CDK 4/6i are scarce and inconsistent (Meattini et al. 2022).

Tumor growth is ruled by local angiogenesis which is inhibited by irradiation. After irradiation, the tumor becomes more hypoxic, and the hypoxia inducible factor-1 (HIF-1) and the stroma-derived factor-1 (SDF-1) are increased as the tumor attempts to regrow. Abemaciclib inhibits restoration of tumor vasculature post irradiation by inhibiting HIF-1 and SDF-1 expression in the tumor microenvironment thereby mitigating radiation-induced vasculogenesis (Naz et al. 2019).

Naz et al. (2018) demonstrated that abemaciclib, in addition to inducing a G1/S phase cell cycle block, inhibited DNA damage repair and mTOR regulation, thus altering amino acid metabolism in vitro. Administering abemaciclib a second week post-radiation/abemaciclib combination to lung cancer xenografts resulted in inhibition of radiation-induced neovascularization, also known as vasculogenesis in vivo and enhanced tumor growth delay. The findings that abemaciclib enhanced tumor cell radiosensitivity, enhanced phosphorylation of gamma-H2AX in combination with radiation, and reduced phosphorylation of p-AKT and p-S6 attenuating PI3K/mTOR signaling as well as alleviating radiation-induced vasculogenesis qualifies it to be a multi-functional radiation modifier.

This work aims to examine the impacts of radiotherapy in combination with the CDK4/6 inhibitor abemaciclib versus abemaciclib administered alone on breast cancer cell lines.

## Material and methods

### Reagents and chemicals

Abemaciclib was purchased from the Lilly Company. High-grade analytical chemicals and reagents were used.

### Cell culture

The human breast cancer cell lines MCF-7, MDA-MB-231, and MDA-MB-468 were afforded frozen in liquid nitrogen at − 180 °C from the American Type Cell Collection (ATCC; Manassas, VA, USA). At the Egyptian National Cancer Institute (NCI) in Cairo, Egypt, they were cultivated in RPMI-1640 supplemented with 10% fetal bovine serum, 100 units/ml penicillin, and 2 mg/ml streptomycin under 5% CO2 at 37 °C. Serial subculturing was used to preserve the tumor cell lines. At a density of 1 × 10^4^ cells/well, the cells were seeded onto plates and incubated for 24 h before the experiments. The National Centre for Radiation Research and Technology’s (NCRRT) research ethics committee in Cairo, Egypt, has approved the treatment protocol (75A/23).

### Irradiation

Using an AECL 137Cs Gamma Cell-40 biological irradiator at the National Centre for Radiation Research and Technology (NCRRT), Egyptian Atomic Energy Authority, Cairo, Egypt, gamma radiation was performed. At a dosage rate of 0.588 cGy/s, cell lines were exposed to gamma irradiation at diverse doses (2, 4, 6, 8, 10 Gy).

### Experimental design

#### Cell culture and radiation exposure

MDA-MB-231 and MDA-MB-486, two breast cancer cell lines that are triple-negative breast cancer (TNBC), as well as MCF-7 mesenchymal transformed cells, were cultured in Dulbecco’s modified Eagle’s medium (DMEM) and Roswell Park Memorial Institute Medium (RPMI1640) from Gibco. These media contain 100 units/mL of penicillin and 100 mg/mL of streptomycin and are supplemented with 10% fetal bovine serum (FBS) from Gibco, then maintained in a humidified 37 °C incubator with 5% CO_2_. For all the experiments, cells were seeded at varying densities and incubated for a full day to achieve 60–70% confluence. Following that, cells were subjected to varying radiation doses (2, 4, 6, 8, and 10 Gy) at a dose rate of 0.588 cGy/s using a biological irradiator (AECL 137Cs Gamma Cell-40) at the Egyptian Atomic Energy Authority in Cairo, Egypt.

#### Cytotoxicity assay

The sulforhodamine-B method was used to determine cytotoxicity (Skehan et al. 1990). The MCF-7, MDA-MB-231, and MDA-MB-486 cells were seeded at a concentration of 3 × 10^3^ cells/well in 96-well microtiter plates and left to attach for 24 h before incubation with different concentrations of abemaciclib for 48 h. Spectrophotometrically, the optical density (O.D.) was measured at 570 nm using an ELISA microplate reader (TECAN Sunrise TM, Germany). The mean data were estimated as the cell viability percentage in the following manner: O.D. (treated cells)/O.D. (control cells) × 100. Using dose–response curve-fitting models, the IC_50_ value (the concentration that results in 50% inhibition of cell growth) of each radiation dose and abemaciclib was determined (GraphPad Prism software, version 5). The best IC_50_ was used for the experiment. According to the results, MCF-7 and MDA-MB-231 were used for the assays. So, MCF-7 and MDA-MB-231 were exposed to different γ-radiation doses (2, 4, 6, 8, and 10) to determine the dose of irradiation that decreased the proliferation of the cells. The chosen dose of irradiation was 10 Gy, so it was used for the experiments.

#### Preparation of cell-free media and cell lysate

Each MCF-7 and MDA-MB-231 cell line was cultured and left for 24 h in T75 flasks. Group I: the control group comprised untreated cells. Group II: cells were treated with the IC_50_ of abemaciclib. Group III: cells were treated with the IC_50_ of abemaciclib and exposed to radiation (10 Gy). All groups were left for 48 h. Trypsinization was used to detach the cells from the flask and prepare cell pellets. The treated and control cell pellets were collected, washed, and suspended in cold lysis buffer, then sonicated and centrifuged, and the clear supernatant was transferred to another Eppendorf.

#### Quantitative real-time polymerase chain reaction (qRT-PCR)

Using quantitative real-time PCR, the gene expression of phosphoinositide 3-kinase (PI3K), serine/threonine-protein kinase (AKT), mammalian target of rapamycin (mTOR), CDK4, CDK6, transforming growth factor-β (TGF-β), and nuclear factor-kappaβ (NF-κβ) (Table 1) in cells was quantified. In 25-mm flasks, 500,000 MCF-7 and MDA-MB-231 cells were seeded overnight. Subsequently, the cells were exposed to either 10 Gy of radiation and 75 μM or 32 μM of abemaciclib for MCF-7 and MDA-MB-231 cells, respectively. Following the manufacturer’s instructions, the miRNeasy Mini Kit (Qiagen, Germany) was used to purify total RNA from treated and untreated cells. A NanoDrop-2000 spectrophotometer (ThermoFisher Scientific, USA) was used. Exploiting the Quantitect RNA reverse transcription kit (Qiagen, Germany) following the producer’s instructions, complementary DNA (cDNA) was synthesized from 1 µg of RNA. On the ViiA™ 7 PCR machine (Applied Biosystems, USA), the relative expression levels of PI3K, AKT, mTOR, CDK-4, CDK-6, TGF-β, and NF-κβ were determined by qPCR using a Quantinova SYBR Green reagent kit from Qiagen with cDNA as the template. As an internal control for mRNAs, B-actin was used. Primers were obtained from Bio Basics. Table 1 lists the primer sequences for each target gene. The data analysis equations utilized were: ∆Ct = Ct (gene of interest) − Ct (housekeeping gene), followed by ∆∆Ct = ∆Ct (treated sample) − ∆Ct (untreated sample). The overall formula was 2^–∆∆C^ to calculate the relative fold of change (Livak and Schmittgen 2001).

**Table 1:** Primer sequences for the real-time quantitative polymerase chain reaction (RT-qPCR)

Gene name	Forward primer	Reverse primer
PI3K	GGTTGTCTGTCAATCGGTGACTGT	GAACTGCAGTGCACCTTTCAAGC
AKT	TTCTGCAGCTATGCGCAATGTG	TGGCCAGCATACCATAGTGAGGT
mTOR	AGCATCGGATGCTTAGGAGTGG	CAGCCAGTCATCTTTGGAGACC
CDK4	GCTGAAATTGGTGTCGGTGC	CACGAACTGTGCTGATGGGA
CDK6	GGTACAGAGCACCCGAAGTC	GCAGCCAACACTCCAGAGAT
TGF-b1	TACCTGAACCCGTGTTGCTCTC	GTTGCTGAGGTATCGCCAGGAA
NF-Kb1	GCAGCACTACTTCTTGACCACC	TCTGCTCCTGAGCATTGACGTC
B-actin	AAAGGGTGGTAACGCAACTA	GGACCTGACTGACTACCTC

#### Estimation of cytokeratin 18 (CK18) and cycloxygenase-2 (COX2)

An enzyme-linked immunosorbent assay (ELISA) kit (Catalog number: CSB-E09172h, Cusabio Biotech Co., China, and Catalog number: MBS264304, My Biosource, respectively) was used to determine the concentrations of CK18 and COX2. The manufacturer’s instructions were followed during this procedure.

#### Pro-inflammatory marker measurement

An ELISA kit (Catalog number: SEA079Ra and Catalog number: MBS175901, My Biosource, respectively) was used to assess the concentrations of interleukins IL6 and IL1β. The procedure was carried out as directed by the manufacturer.

#### Apoptotic marker estimation

B-cell lymphoma protein 2 (Bcl-2)-associated X (Bax), B-cell lymphoma protein 2 (Bcl-2), and tumor protein P53 (P53) were determined using an ELISA kit (catalog numbers: SEB343Mu, SEA778Ra, and SEH009Hu (Cloud-Clone Corp., USA; respectively). Human caspases 3, 6, 7, 8, and 9 were analyzed using ELISA Kits; human caspase-3, caspase-6 (catalog number: SEA626Hu, SEC340Hu; respectively), Cloud-Clone Corp, USA; human caspase-7 (catalog Number: CSB-EL004552HU) Cusabio Biotech Co., China; human caspase-8 (catalog number: MBS452285) My Biosource; human caspase-9 (catalog number: ab119508). The procedure was carried out as directed by the manufacturer.

#### Matrix metalloproteinase (MMP2 and MMP9) estimation

MMP2 and MMP9 were analyzed using ELISA Kits (catalog number: MBS824931 and MBS175780; respectively) My Biosource following the manufacturer’s instructions.

#### Oxidative stress marker measurement

Using the Buege and Aust technique (Buege and Aust 1978), the amount of malondialdehyde (MDA) in the cell lysate was estimated to assess the lipid peroxidation level. Spectrophotometrically, nitric oxide (NO) and reduced glutathione (GSH) levels in cell lysate were estimated using the methods of Miranda et al. (2001) and Ellman (1959); respectively.

### Statistical analysis

GraphPad Prism was used for statistical calculations. All values were presented as mean ± SEM. Statistical analysis was performed using two-way analysis of variance (ANOVA) for the cytotoxicity assay and one-way ANOVA for the rest of experiments. Tukey’s post hoc test was used for multiple group comparisons. Values with *p* < 0.05 were considered to be statistically significant.

## Results

### Cytotoxicity of abemaciclib on MCF-7, MDA-MB-231, and MDA-MB-468 breast cancer cell lines (A) and effect of different radiation doses on cell viability of MCF-7 and MDA-MB-231 breast cancer cell lines (B)

**Fig. 1:**
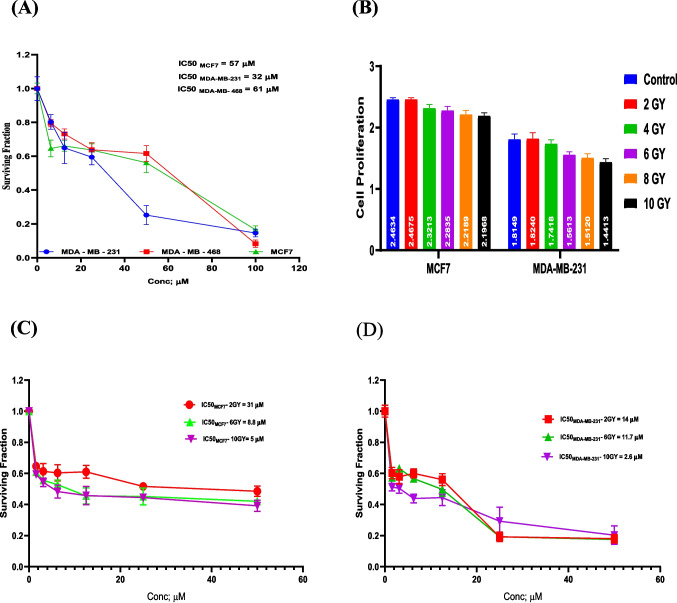
Cytotoxicity of abemaciclib (6.25, 12.5, 25, 50 and 100 μM) on MCF-7, MDA-MB-231, and MDA-MB-468 breast cancer cell lines (**A**), effect of different doses of radiation (2, 4, 6, 8, and 10 Gy) on cell proliferation of MCF7 and MDA-MB-231 (**B**) and cytotoxicity of abemaciclib (6.25, 12.5, 25, 50 and 100 μM) on MCF-7 and MDA-MB-231 exposed to different radiation doses (2, 6, and 10 Gy) and evaluated 48 hours after the irradiation (**C** and **D**), values are represented as the means ± SD of three experiments (each performed in triplicate). The data represent the mean ± standard deviation (SD) of three experiments performed in triplicate

Figure 1 epitomized that abemaciclib showed anticancer activity on MCF-7, MDA-MB-231, and MDA-MB-486 cell lines with IC_50_ equivalents to 57, 32, and 61 μM, respectively (Fig. 1A). According to these results, MCF-7 and MDA-MB-231 cell lines were used for the rest of the assays. Exposure of MCF-7 and MDA-Mb-231 to different doses of irradiation showed that irradiation (10 Gy) had the best IC_50_ on the MCF-7 and MDA-MB-231 cell lines (Fig. 1B).

#### Effect of treatment with abemaciclib and irradiation on the gene expression of CDK4, CDK6, TGF-β1, and NF-κβ in the cell lysate of MCF-7 and MDA-MB-231 cell lines

**Fig. 2:**
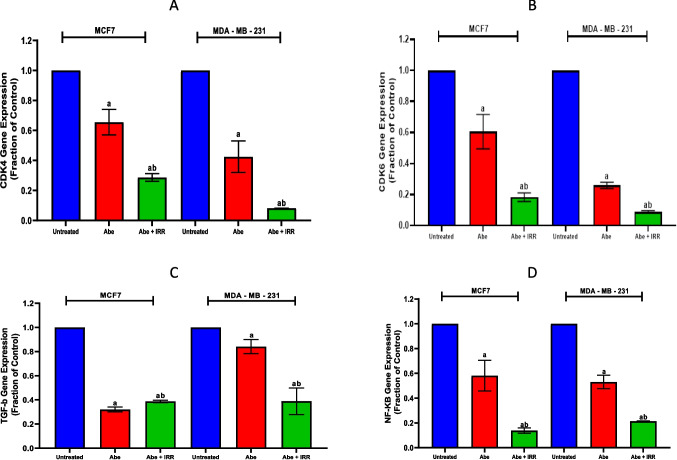
Effect of treatment with abemaciclib (Abe) and irradiation (IRR, 10 Gy) on the gene expression of CDK4, CDK6, TGF-b1, and NF-kB in the cell lysate of MCF-7 and MDA-MB-231 cell lines. CDK4 gene expression (**A**), CDK6 gene expression (**B**), TGF-b1 gene expression (**C**), and NF-kB gene expression (**D**). Results are expressed as means ± SD of two independent experiments performed in triplicate. The statistical significance of the results was analyzed using a one-way ANOVA followed by Tukey’s multiple comparison test. (a) Significantly different from the untreated cells; (b) Significantly different from Abe at *P* < 0.05

The gene expressions of CDK4, CDK6, TGF-β, and NF-κβ were significantly reduced by abemaciclib in breast cancer cell lines MCF-7 and MDA-MB-231 compared to untreated cells of each cell line. In MCF-7 and MDA-MB-231 cell lines, the combination of abemaciclib with IRR (10 Gy) incited a significant diminution in the gene expressions of CDK4, CDK6, TGF-β1, and NF-κβ comparable to abemaciclib-treated cells (Fig. 2).

#### Effect of abemaciclib and irradiation on the gene expression of PI3K, AKT, and mTOR in the cell lysate of MCF-7 and MDA-MB-231 cell lines

**Fig. 3:**
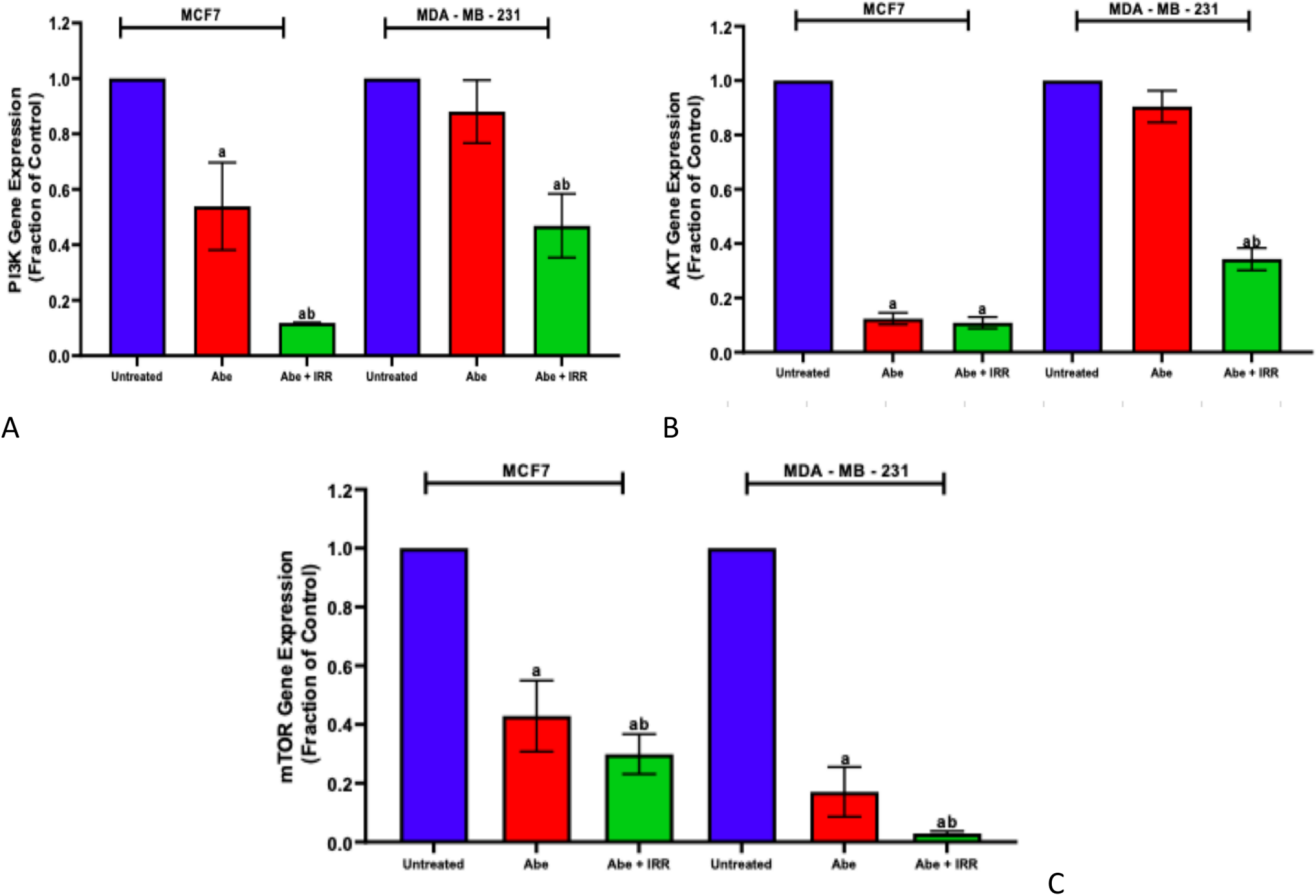
Effect of abemaciclib (Abe) and irradiation (IRR, 10 Gy) on the gene expression of PI3K, AKT, and mTOR in the cell lysate of MCF-7 and MDA-MB-231 cell lines. PI3K gene expression (**A**), AKT gene expression (**B**), and mTOR gene expression (**C**). Results are expressed as the means ± SD of two independent experiments performed in triplicate. The statistical significance of the results was analyzed using a one-way ANOVA followed by Tukey’s multiple comparison test. (a) Significantly different from the untreated cells; (b) Significantly different from Abe at *P* < 0.05

Compared to untreated cells, abemaciclib expressively diminished the expression of the PI3K, AKT, and mTOR genes in the MCF-7 cell line. On the other hand, in the MDA-MB-231 cell line, abemaciclib induced a non-significant decrease in the gene expression of PI3K, and AKT, and significant decrease in mTOR gene expression comparable to the untreated cells (Fig. 3). The grouping of abemaciclib and IRR induced a significant diminution in the PI3K and mTOR gene expression compared to abemaciclib-treated cells in MCF-7 and MDA-MB-231 cell lines. Furthermore, the grouping of abemaciclib and IRR induced a significant decrease in AKT gene expression compared to abemaciclib-treated cells in the MDA-MB-231 cell line and no change in the MCF-7 cell line (Fig. 3).

#### Effect of treatment with abemaciclib, irradiation, and their combinations on the concentration of BAX, Bcl-2, and P53 in the cell lysate of MCF-7 and MDA-MB-231 cell lines

**Fig. 4:**
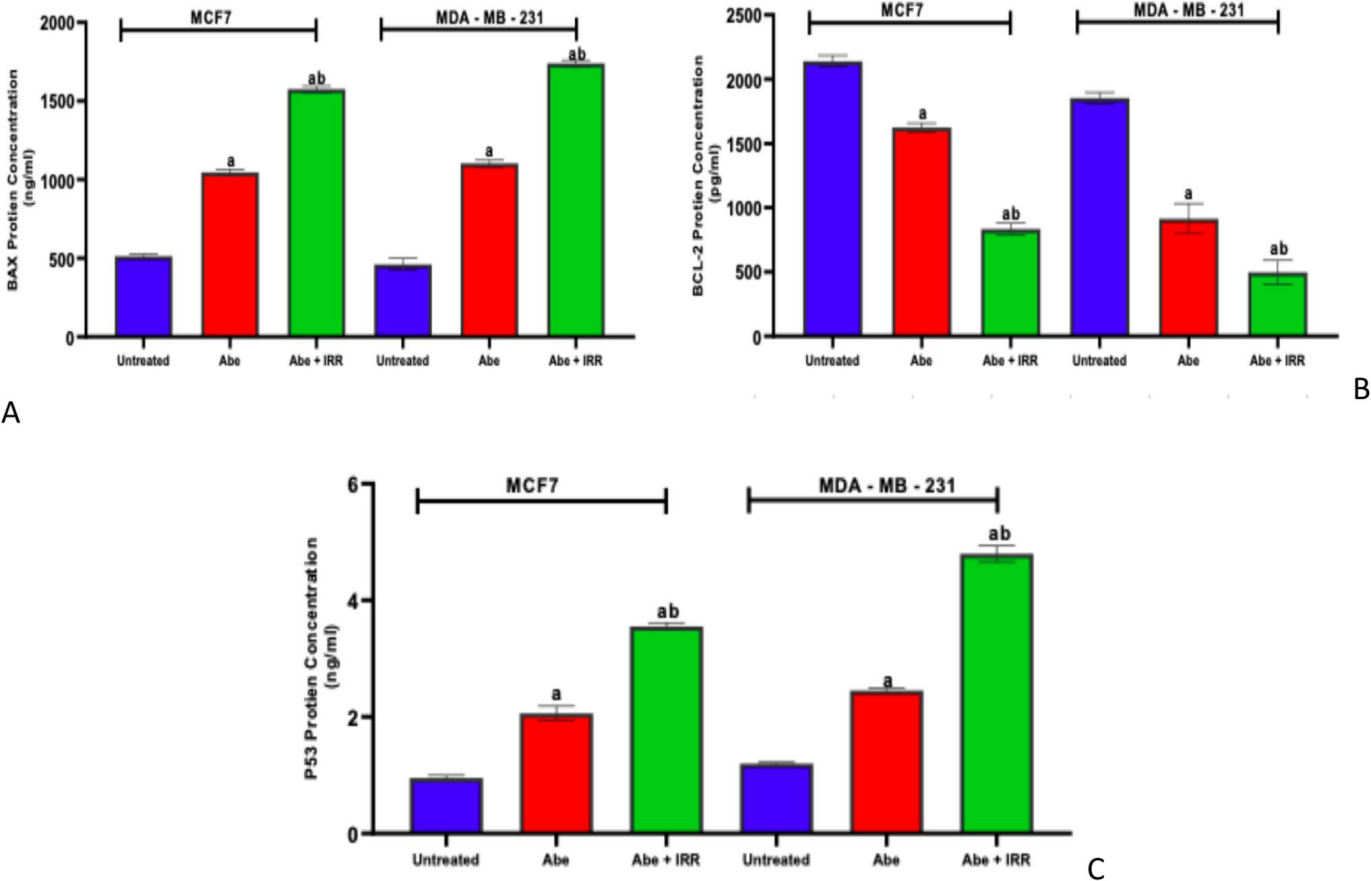
Effect of treatment with abemaciclib (Abe), irradiation (IRR, 10 Gy), and their combinations on the concentration of BAX, Bcl-2, and P53 in the cell lysate of MCF-7 and MDA-MB-231 cell lines. BAX concentration (**A**), Bcl-2 concentration (**B**), and P53 concentration (**C**). Results are expressed as means ± SD of two independent experiments performed in triplicate. The statistical significance of the results was analyzed using a one-way ANOVA followed by Tukey’s multiple comparison test. (a) Significantly different from the untreated cells; (b) Significantly different from Abe at *P* < 0.05

Abemaciclib significantly boosted the protein concentration of BAX and P53 and significantly reduced Bcl-2 in MCF-7 and MDA-MB-231 cell lines compared to untreated cells. On the other hand, abemaciclib and IRR (10Gy) significantly increased the concentration of BAX and P53 and significantly decreased Bcl-2 in MCF-7 and MDA-MB-231 cell lines compared to abemaciclib-treated cells (Fig. 4).

#### Effect of treatment with abemaciclib and irradiation on the concentration of caspase-3 (Fig. 5A), caspase-6 (Fig. 5B), caspase-7 (Fig. 5C), caspase-8 (Fig. 5D), and caspase-9 (Fig. 5E) in the cell lysate of MCF-7 and MDA-MB-231 cell lines

**Fig. 5:**
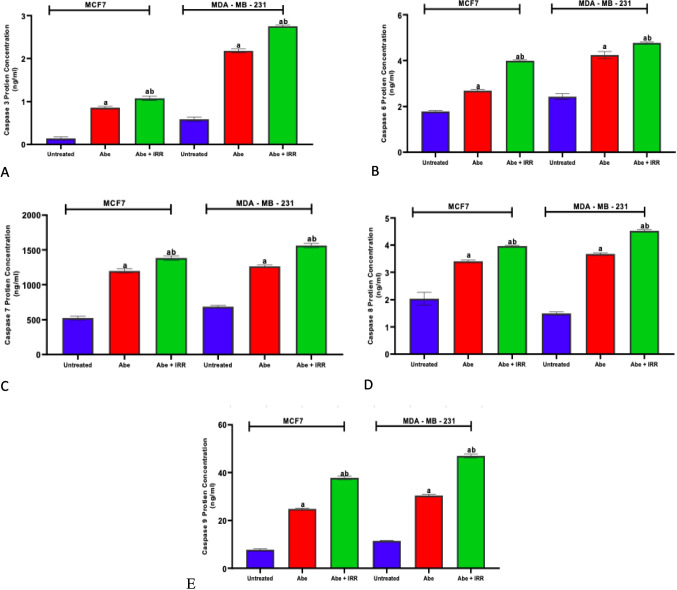
Effect of treatment with abemaciclib (Abe) and irradiation (IRR, 10 Gy) on the concentration of caspase-3 (**A**), caspase-6 (**B**), caspase-7 (**C**), caspase-8 (**D**), and caspase-9 (**E**) in the cell lysate of MCF-7 and MDA-MB-231 cell lines. Results are expressed as means ± SD of two independent experiments performed in triplicate. The statistical significance of the results was analyzed using a one-way ANOVA followed by Tukey’s multiple comparison test. (a) Significantly different from the untreated cells; (b) Significantly different from Abe at *P* < 0.05

In comparison to the untreated cells of MCF-7 and MDA-MB-231 cell lines, Fig. 5 shows that abemaciclib significantly increased the amounts of caspase-3, caspase-6, caspase-7, caspase-8, and caspase-9. The concentrations of caspase-3, caspase-6, caspase-7, caspase-8, and caspase-9 were significantly higher in MCF-7 and MDA-MB-231 cell lines treated with abemaciclib and IRR (10 Gy), compared to abemaciclib-treated cells (Fig. 5A–E).

#### Effect of treatment with abemaciclib and irradiation on the concentration of CK18, COX2, IL-6, and IL-1β in the cell lysate of MCF-7 and MDA-MB-231 cell lines

**Fig. 6:**
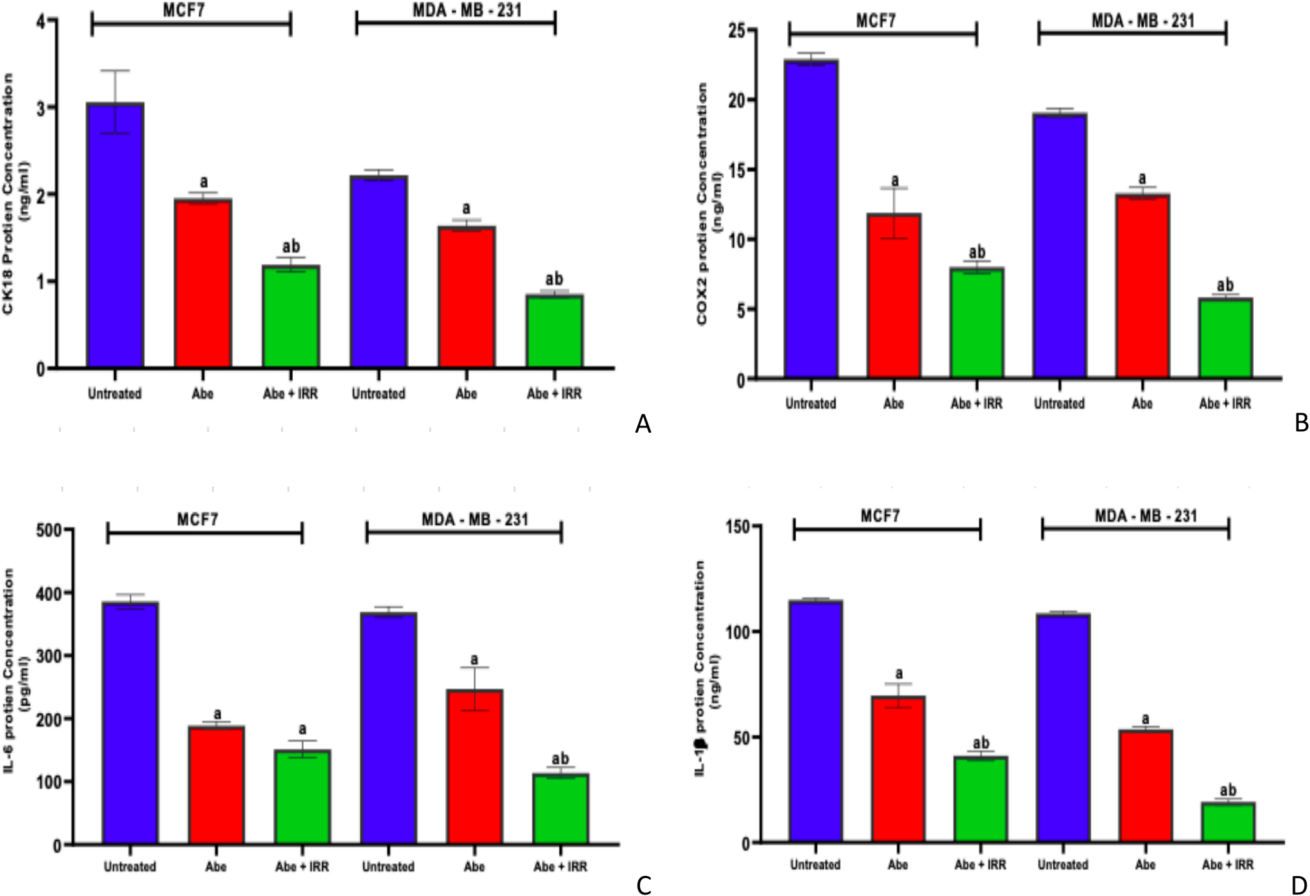
Effect of treatment with abemaciclib (Abe) and irradiation (IRR, 10 Gy) on the concentration of CK18, COX2, IL-6, and IL-1b in the cell lysate of MCF-7 and MDA-MB-231 cell lines. CK18 concentration (**A**), COX2 concentration (**B**), IL-6 concentration (**C**), and IL-1b concentration (**D**). Results are expressed as means ± SD of two independent experiments performed in triplicate. The statistical significance of the results was analyzed using a one-way ANOVA followed by Tukey’s multiple comparison test. (a) Significantly different from the untreated cells; (b) significantly different from Abe at *P* < 0.05

Figure 6 demonstrates that regarding the untreated cells of each cell line, abemaciclib significantly reduced the levels of CK18, COX2, IL-6, and IL-1β in the cell lysate of MCF-7 and MDA-MB-231. Abemaciclib and IRR (10 Gy) induced a significant reduction in the level of CK18 (Fig. 6A), COX2 (Fig. 6B), and IL-1β (Fig. 6D), in the cell lysate of MCF-7 and MDA-MB-231 cell lines compared to the abemaciclib-treated cells of each cell line. Furthermore, abemaciclib and IRR (10 Gy) induced a non-significant decrease in the level of IL-6 in the cell lysate of MCF-7 and a significant reduction in the level of IL-6 in MDA-MB-231 cell lines compared to the abemaciclib-treated cells of each cell line (Fig. 6C).

#### Effect of treatment with abemaciclib and irradiation on the concentration of glutathione (GSH) content, malondialdehyde (MDA), and nitric oxide (NO) levels in the cell lysate of MCF-7 and MDA-MB-231 cell lines

**Fig. 7:**
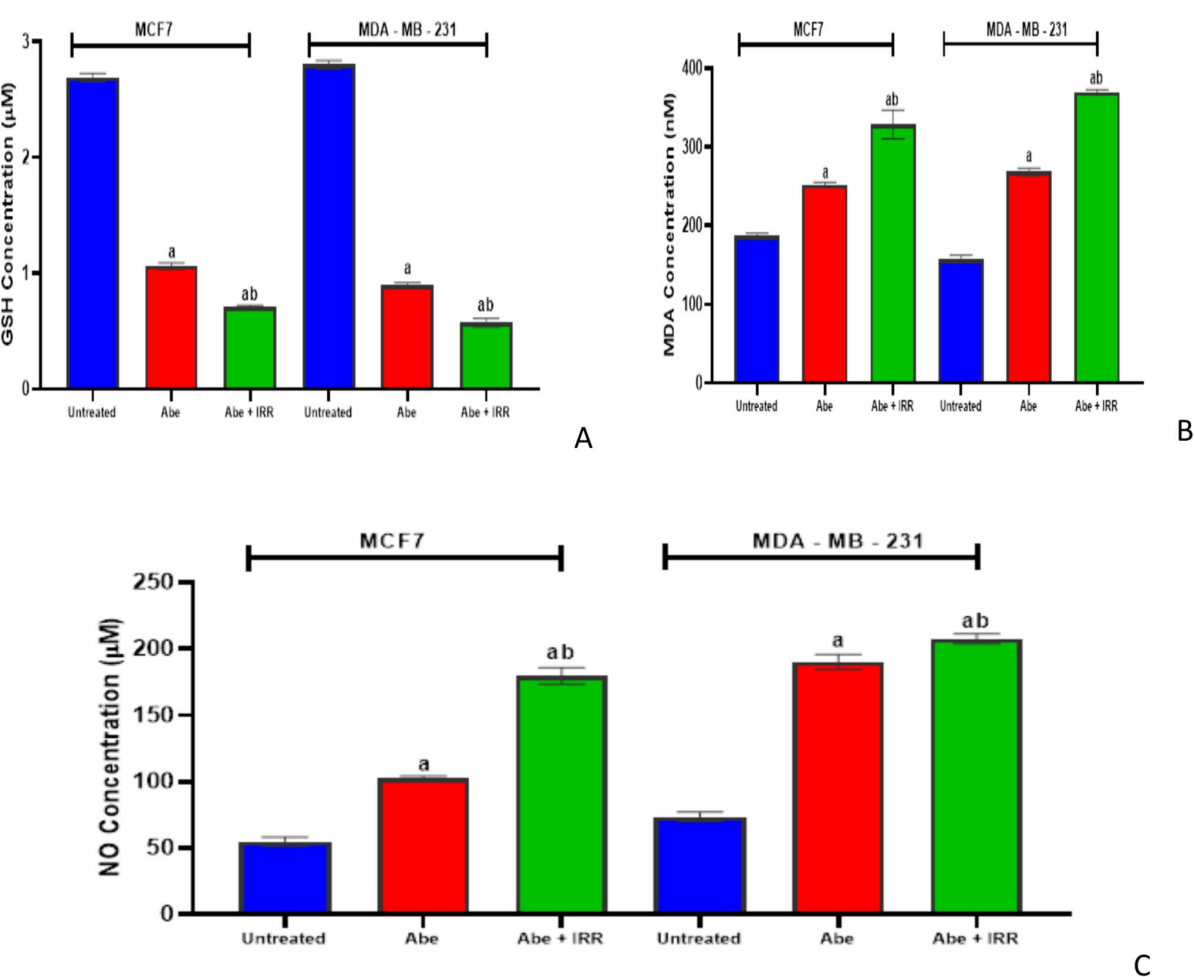
Effect of treatment with abemaciclib (Abe) and irradiation (IRR, 10 Gy) on the concentration of glutathione (GSH) content, malondialdehyde (MDA), and nitric oxide (NO) levels in the cell lysate of MCF-7 and MDA-MB-231 cell lines. GSH concentration (**A**), MDA concentration (**B**), and NO concentration (**C**). Results are expressed as means ± SD of two independent experiments performed in triplicate. The statistical significance of the results was analyzed using a one-way ANOVA followed by Tukey’s multiple comparison test. (a) Significantly different from the untreated cells; (b) significantly different from Abe at *P* < 0.05

Figure 7 shows that abemaciclib triggered a significant increase in the levels of MDA and NO in comparison to the untreated cells of MCF-7 and MDA-MB-231 cell lines, while GSH content decreased significantly in comparison to the untreated cells of MCF-7 and MDA-MB-231 cell lines. There was an apparent significant increase in the levels of MDA and NO and a significant reduction in GSH in MCF-7 and MDA-MB-231 cell lines treated with abemaciclib and IRR (10 Gy), compared to abemaciclib-treated cells (Fig. 7A–C).

#### Effect of treatment with abemaciclib and irradiation on the matrix metallopeptidase 2 and 9 (MMP2 and MMP9) levels in the cell lysate of MCF-7 and MDA-MB-231 cell lines

**Fig. 8:**
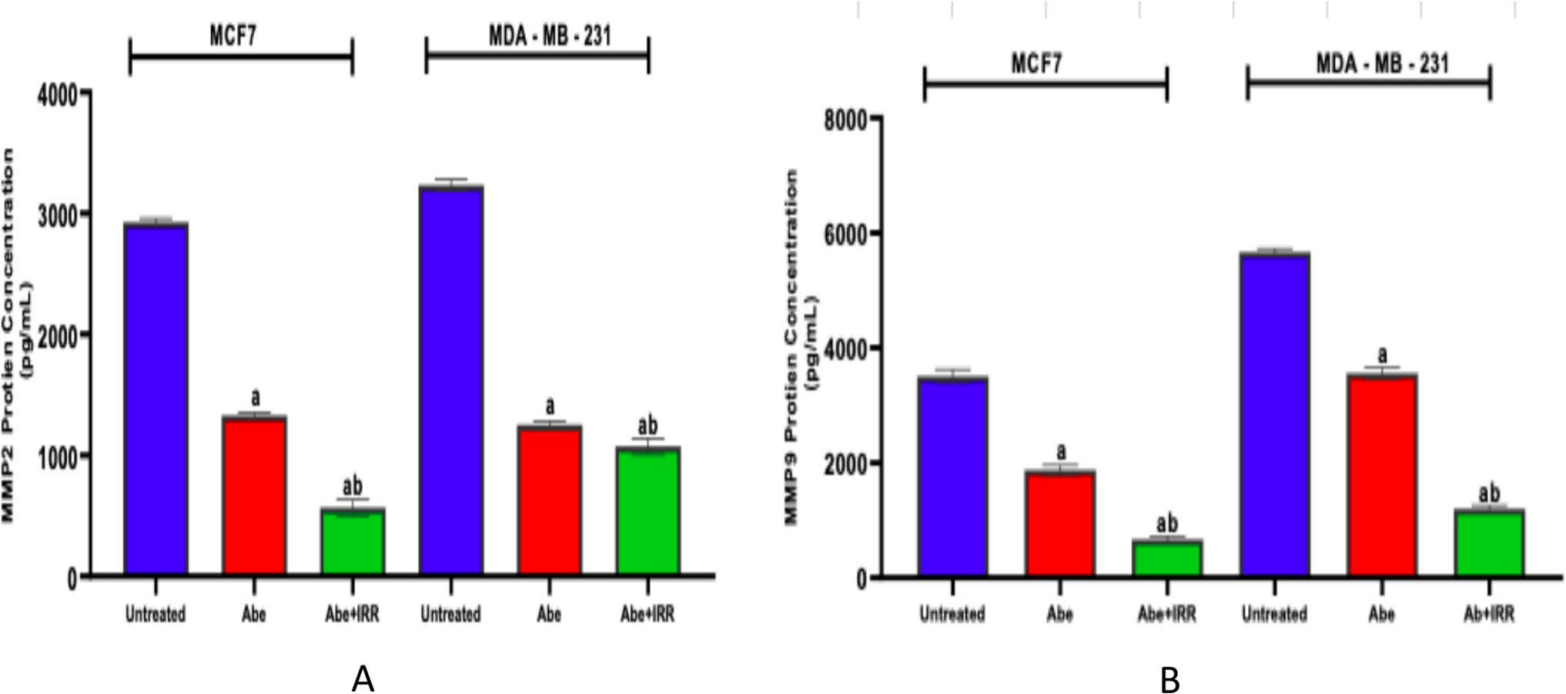
Effect of treatment with abemaciclib (Abe) and irradiation (IRR, 10 Gy) on the matrix metallopeptidase 2 and 9 (MMP2 and MMP9) levels in the cell lysate of MCF-7 and MDA-MB-231 cell lines. MMP2 (**A**), MMP9 (**B**). Results are expressed as the means ± SD of two independent experiments performed in triplicate. The statistical significance of the results was analyzed using a one-way ANOVA followed by Tukey’s multiple comparison test. (a) Significantly different from the untreated cells; (b) Significantly different from Abe at *P* < 0.05

Figure 8 shows that abemaciclib instigated a significant decrease in the levels of MMP2 and MMP9 in comparison to the control cells of MCF-7 and MDA-MB-231 cell lines. There was a significant decrease in the levels of MMP2 and MMP9 in the MCF-7 and the MDA-MB-231 cell lines treated with abemaciclib and irradiation (IRR, 10 Gy) compared to abemaciclib-treated cells (Fig. 8A, B).

## Discussion

Serine/threonine kinases called CDKs are controlled by their interactions with cyclins and CDKi. The quelling of the cyclin/CDK4/6 complex by abemaciclib has shown promise in the treatment of hormone-sensitive and TNBC patients. The mechanisms underlying the combined effects of radiotherapy and CDK4/6 inhibitors remain unknown. To reach this goal, we used one ER + and two TNBC cell lines. Among the three tested breast cancer cell lines, MDA-MB-231 and MCF-7 cells displayed the best IC50. However, the results demonstrated that cytotoxicity was significantly decreased at 48 h after irradiation exposure at 10 Gy in both cell lines.

In preclinical investigations, CDK4/6 inhibitors have enhanced apoptosis, caused cellular senescence, reduced DNA damage repair, and arrested the cell cycle, all of which have radiosensitized cancer cells (Franco et al. 2023). In a metastatic situation, RT has been known for its safety and is regarded as a crucial module of palliative therapy, mainly in the relief of bone pain instigated by symptomatic lesions in cancer patients (Becherini et al. 2023).

In the undercurrent study, abemaciclib induced a distinct diminution in the expression of the PI3K/AKT/mTOR pathway and CDK4, CDK6, and TGF-β1/NF-κβ in MCF-7 and MDA-MB-231 cell lines, whereas the expression of the PI3K/AKT/mTOR pathway, CDK4, CDK6, and TGF-b1/NF-κβ was more decreased by the grouping of abemaciclib and IRR (10 Gy). The gathering of abemaciclib and IRR (10 Gy) significantly increased the protein concentration of BAX and P53 and decreased Bcl-2, CK18, COX2, IL-6, and IL-1β concentrations in MCF-7 and MDA-MB-231 cell lines emulated to abemaciclib alone.

Abemaciclib regulates the succession of the cell cycle by urging the development of the cyclin D-CDK4/6 complex, which specifically inhibits the downstream CDK4/6-mediated phosphorylation of Rb, instigating cell cycle arrest in the G0/G1 phase (Piezzo et al. 2020; George et al. 2021). Additionally, in a dose-dependent mode, abemaciclib slowed the growth of HPV-negative cervical cancer cells and prostate cancer cells. It also induced apoptosis by suppressing the CDK4/6-Cyclin D complex, activating NFκβ through the degradation of Iκβ and mTOR pathways, producing ROS, depolarizing the mitochondrial membrane potential, and overexpressing proapoptotic genes (Wagner and Gil 2020; Liu et al. 2021; Eskiler et al. 2022).

In agreement with the current investigation, hormone-sensitive and TNBC cell proliferation is markedly inhibited by RT and/or abemaciclib therapy by inducing apoptosis.

Compared to CDK 4/6 inhibition alone, preclinical research showed that CDK 4/6 inhibition during or after RT induced DNA double-strand break repair and enhanced tumor cell death (O'Brien et al. 2018; Kim et al. 2021b). Moreover, other inflammatory pathways are rendered inactive as a result of the production of DNA damage during this phase (Riley et al. 2018).

In settlement with preceding studies (Liu et al. 2021; Eskiler et al. 2022), abemaciclib treatment instigated a significant increment in the rate of apoptosis by quelling CDK4/6, the overexpressing caspase-3, caspase-6, caspase-7, caspase-8, and caspase-9 levels, and downregulating the PI3K/AKT/mTOR pathway in MCF-7 and MDA-MB-231 cell lines. A significant augmentation in BAX and P53 and a significant diminution in BcL2 were distinguished in MDA-MB-231 and MCF-7 cells following RT and/or abemaciclib. In coincidence with the undercurrent study, Lin et al. (2013) conveyed that irradiation augmented apoptosis markers, lessened Bcl-2 expression, and amplified BAX and P53 expression in breast cancer cell lines.

The overexpressions of caspase-3, caspase-6, caspase-7, caspase-8, and caspase-9 reported in the present study proposed that both the intrinsic and extrinsic pathways of apoptosis were associated with RT and/or abemaciclib in MCF-7 and MDA-MB-231 cells.

Apoptosis, a form of programmed cell death, is induced by two different pathways, intrinsic and extrinsic; both pathways complete apoptosis by initiating the execution pathway. Microenvironmental disturbances such as DNA damage, endoplasmic reticulum stress, reactive oxygen species (ROS) excess, microtubular changes, and mitotic abnormalities can cause intrinsic apoptosis. Ionizing radiation can cause permanent changes to the microenvironment of MCF-7 cells in several ways, mainly because it can act on the mitochondrial membranes and endoplasmic reticulum to trigger apoptotic signals. Extrinsic apoptosis is a type of cell death that is triggered by the disturbances of the extracellular microenvironment by plasma membrane receptors and activates the decisive execution pathway mediated by caspase-8 (Jiao et al. 2022).

The protein families Bcl-2 and caspase are involved in the organization of apoptosis. The permeability of the mitochondrial outer membrane is regulated by interactions between the Bcl-2 family proteins. Cytoplasmic proteins B-cell lymphoma protein 2 (Bcl-2)-associated X (Bax) and Bcl-2 act as promoters and inhibitors of apoptosis, respectively (Kulsoom et al. 2018). Upon DNA damage, endoplasmic reticulum stress, hypoxia, or free radicals, the activity of (tumor protein P53) p53 is directed toward cell death through the initiation of Bcl-2 gene expression in addition to direct interactions with Bcl-2 family proteins (Chen 2016; Aubrey et al. 2018). It has been demonstrated that Bax overexpression in ER + breast cancer cells requires p53 activation (Kawiak and Kostecka 2022). Bax protein stimulates the cascade of reactions inducing apoptosis by emancipating cytochrome c from the mitochondria, which triggers caspases and ultimately performs cell death (Brentnall et al. 2013).

Apoptotic caspases are categorized as initiator caspases (caspase-8, caspase-9, and caspase-10) and effector caspases (caspase-3, caspase-6, and caspase-7) (Man and Kanneganti 2016). By releasing cytochrome c and activating caspase-9 and caspase-8 initiators through mitochondrial outer membrane permeabilization (MOMP), pro- and anti-apoptotic BcL2 family members excellently synchronize mitochondrial membranes (Galluzzi et al. 2016). Once activated, caspase-8 and caspase-9 cleave and trigger caspase-3 and caspase-7, resulting in the execution of apoptosis (Orning and Lien 2021; Kawiak and Kostecka 2022). However, in MCF7 cells, Blanc et al. (2000) reported that cytochrome c- and caspase-8-mediated processing of procaspase-9 is strictly dependent on caspase-3, suggesting that caspase-3 may be precarious for the regulation of procaspase-9. Activation of caspase-9 and caspase-3 restrains ROS production and is requisite for efficient execution of apoptosis, while effector caspase-7 is required for apoptotic cell detachment (Brentnall et al. 2013).

In the early stages of dissimilar types of solid tumors, depending on the type of cancer and the size and location of the tumor, RT is a successful treatment that is used as a palliative treatment in metastatic phases (Halkett et al. 2018). Both directly and indirectly, ionizing radiation damages DNA, instigating the ionization of the atoms, breaking the bonds between the atoms in DNA molecules, and the production of highly reactive free radicals, which can interact with the DNA. Nevertheless, the damage is sensed by tumor cells through a DNA damage response mechanism that stimulates the activation of cell cycle checkpoints, induces cell cycle arrest, and activates apoptosis (Huang and Zhou 2020). Radiation caused apoptosis in a glioma U251 cell line by expressing caspase-3 and Bax (Carlos-Reyes et al. 2021). Ethylcellulose-based nanosponges loaded with abemaciclib maintained their cytotoxicity against MCF-7 and MDA-MB-231 human breast cancer cell lines (Anwer et al. 2022).

Previous studies suggested that abemaciclib induces oxidative stress via triggering endoplasmic reticulum stress, changing the mitochondrial membrane potential, inducing mitochondrial-dependent apoptosis, activating JNK/MAPK, and inhibiting PDGFR-mediated PI3K/AKT signaling (Riley et al. 2018; Eskiler et al. 2022). PI3K is a family of lipid kinases that phosphorylate phosphatidylinositol at the intracellular membrane and plasma membranes. An increase in PI3K/AKT/mTOR signaling has been documented, contributing to proliferation via the NF-κβ pathway, metastasis, and drug resistance in breast cancer cells (George et al 2021; Saha and Lukong 2022). Inhibition of mTOR principally leads to activation of downstream protein kinases mandatory for ribosomal biosynthesis and interruption of mRNA translation, resulting in the failure of the cell-cycle transition from the G1 to the S phase (Li et al. 2023).

In the current study, abemaciclib induced a significant decrease in the expression of PI3K, AKT, and mTOR. PI3K/mTOR inhibitors induce synergistic anti-proliferative and pro-apoptotic effects by inhibiting the kinase activity of the CDK/cyclin complex and, consequently, preventing phosphorylation of the Rb protein (Cretella et al. 2018; Jayanthan et al. 2022).

Abemaciclib suppresses cervical cancer cell, pancreatic ductal adenocarcinoma, prostate cancer, and TNBC cell growth by promoting apoptosis via the suppression of CDK4/6-Rb-E2F and mTOR pathways, overexpression of caspase-3, pro-apoptotic proteins, and cell cycle regulatory proteins (p53), as well as downregulation of apoptosis 2 inhibitor, X-linked inhibitor of apoptosis protein, and heat shock protein 60 (Eskiler et al. 2022; Alian et al. 2024). It was reported that abemaciclib caused such a cell death phenotype in prostate cancer cells, A549 non-small lung cancer carcinoma cells, and MDA-MB-231 triple-negative breast cancer cells (Hino et al. 2020). Naz et al. (2018) demonstrated that abemaciclib, in addition to inducing a G1/S phase cell cycle block, inhibited DNA damage repair and mTOR regulation, thus altering amino acid metabolism in vitro.

The grouping of RT and abemaciclib induced a significant diminution in the expression of PI3K, AKT, and mTOR compared to abemaciclib alone. Samani et al. (2020) reported that RT, along with trastuzumab, lessened Akt and mitogen-activated protein kinase/extracellular regulated kinase (MAPK/ERK) phosphorylation and boosted apoptosis compared to trastuzumab alone in breast cancer cell lines. The mTOR signaling pathway is the most closely connected pathway to PI3K inhibition. However, inhibition of PI3K by RT also affects the expression of other pathways that boost the efficacy of radiation treatment, such as NF-κβ signaling or ERK/MEK signaling (Pesch et al. 2021).

Radiation has been shown to damage tumor vasculature and inhibit angiogenesis (Barker et al. 2015). Vasculogenesis is the process that leads to tumor blood vessel regeneration and, thus, recurrence after radiation therapy (Naz et al. 2019). The findings indicate that abemaciclib, in combination with RT, enhanced tumor cell radiosensitivity, enhanced gamma-H2AX phosphorylation, reduced AKT phosphorylation, attenuated PI3K/mTOR signaling, alleviated radiation-induced vasculogenesis, and enhanced tumor growth delay (Naz et al. 2019).

The transforming growth factor-β (TGF-β) signaling pathway is hyperactivated in breast cancer, which promotes the cancer progression and metastasis. It controls numerous essential processes, such as cellular growth, differentiation, apoptosis, extracellular matrix synthesis, angiogenesis, and immune responses (Aggarwal et al. 2021; Kim et al. 2021a). MCF-7 breast cancer cells in a TGFβ-dependent EMT program exhibited an increased expression of TGF-b, matrix MMP2, and connective tissue growth factor (CTGF) (Ziegler et al. 2014). CTGF mRNA expression is upregulated in MDA-MB-231 and MCF-7 (Hellinger et al. 2020), mediates numerous downstream actions of TGF-β, and participates in extracellular matrix remodeling (Tsai et al. 2018). The dysregulation of the TGF-β pathway could contribute to tumorigenesis by affecting cancer proliferation, progression, EMT, and metastasis (Li et al. 2024).

NF-κβ plays an essential role in the controlling of inflammation, proliferation, and the survival of cell lines. NF-κβ is regulated by recognized signaling pathways (triggered by IL-1, TNF-α, ROS, and LPS) and noncanonical signaling pathways, which are triggered by inflammatory stimuli across IKKα. To reduce the amount of ROS in tumor cells, which ultimately perform the function of apoptosis inhibition, NF-κβ may upregulate anti-apoptotic molecules like Bcl-XL and Bcl-2 and promote the anti-oxidant enzyme transcription (Wu et al. 2023). Thus, activated NF-κβ is detected in various cancers (Pavitra et al. 2023). Previous studies conveyed that NF-κβ can dramatically increase the expression of many genes in inflammatory breast cancer, including CD40, IL15, COX2, and CXCL1. Furthermore, COX2 and CXCL1 are upregulated in MBC (Devanaboyina et al. 2022; Pavitra et al. 2023). Consistent with this, the combination of RT and abemaciclib decreased the expression of TGF-β1/NF-κβ in MDA-MB-231 and MCF-7 cell lines, which could potentiate apoptosis and further hinder the proliferation of breast cancer cells.

IL-1β and IL-6 expression is increased in the early stages of breast malignancies and is positively correlated with advanced tumor stages, indicating a critical role in breast tumor metastasis (Manore et al. 2022). The current study demonstrated that RT and/or abemaciclib decreased IL-1β and IL-6 concentrations in MDA-MB-231 and MCF-7 cell lines, indicating the antiproliferative effect of RT and abemaciclib. A previous study demonstrated that in inflammatory breast cancer, decreasing NF-κβ significantly suppresses the expressions of IL-1β and IL-6 (Pavitra et al. 2023).

Abemaciclib induced a significant diminution in GSH, as well as a distinct increment in MDA and NO levels indicating a diminishing in oxidative stress defense. In agreement with earlier research, our findings demonstrated that abemaciclib triggers the endoplasmic reticulum (ER) stress response, modifies the mitochondrial membrane potential, and consequently causes loss of membrane integrity, cytochrome c release, ROS generation, cellular endogenous antioxidant depletion, caspase activation, pro-apoptogenic proteins BAX and P53 upregulation, and anti-apoptogenic protein Bcl-2 downregulation. These events culminate in cellular apoptosis (Riley et al. 2018; Eskiler et al. 2022).

A class of zinc endopeptidases known as matrix metalloproteinases (MMPs) is important for breaking down the basement membrane and extracellular matrix (ECM) components (Jiang and Li 2021). The overproduction of MMP2/9 in cancer leads to the degradation of ECM, allowing tumor cells to invade other tissues and spread through metastasis and cell death, proliferation, and angiogenesis (Deng et al. 2019; Zhu et al. 2019; Jiang and Li 2021). MMP2-selective inhibitors were shown to prevent bone metastasis in breast cancer (Li et al. 2023). In the present study, the poor prognosis of MDA-MB-231 and MCF-7 breast cancer cell lines is indicated by the inhibition of MMP2 and MMP9 by RT and/or abemaciclib. Abemaciclib’s anti-migratory actions may be attributed to its ability to decrease NF-κβ and enhance the levels of active Rb protein. NF-κβ directly activates MMP-9, which breaks down the ECM and promotes cell migration to the lymph nodes and/or circulation (Alian et al. 2024).

Cytokeratin 18 (CK18), a type I cytokeratin of the intermediate filament family, is essential for withstanding external cellular stresses and maintaining cellular structural integrity. CK18 regulates cellular processes like mitosis, apoptosis, and proliferation (Yang et al. 2018; Wu et al. 2023). Ha et al. (2012) revealed that a bad prognosis for breast cancer might be inferred from a loss of CK18 expression. The development of chemoresistance, metastasis, and tumor recurrence in breast cancer is due to the induction of cancer stem cells and epithelial-mesenchymal transition (EMT). Downregulation of CK18 is a serious molecular consequence in EMT. Through the Wnt/β-catenin pathway, depletion of CK18 facilitated partial EMT and the acquisition of stemness features in MCF-7 breast cancer cells (Shi et al. 2020). It has been revealed that TGF-β can cause a partial EMT condition and enhance the stemness potential and migratory/invasive capacity of hepatocellular carcinoma cells (Li et al. 2024). In breast epithelial cells, reduced CK18 expression is important for the instigation of TGF-β1-induced EMT (Li et al. 2024). According to our research, CK18 downregulation induced by RT and/or abemaciclib may be important in increasing the apoptotic response of breast cancer cell lines and directing its therapeutic potential toward addressing the disease’s metastasis and progression.

Cycloxygenase-2 (Cox-2) catalyzes the metabolic switch of arachidonic acid to prostaglandins, which inspires Cox-2’s pro-inflammatory and tumor-promoting actions. Additionally, Cox-2 activation can surge the malignant cells’ resistance to radiotherapy. Cancers of the breast, thyroid, ovaries, colon, lung, and prostate have all been shown to overexpress Cox-2. Reduced colon cancer cell apoptosis and increased production of molecules that promote angiogenesis have been associated with overexpression of Cox-2. Cox-2 inhibition inhibits the growth of polyps, lowers the production of proangiogenic factors, and restores apoptosis (Alian et al. 2024). The concept that abemaciclib causes apoptosis and raises cell radiosensitivity is supported by the reduction in Cox-2 in breast cancer cells.

## Conclusion

Abemaciclib hindered MCF-7 and MDA-MB-231 breast cancer cell growth and enhanced RT by inducing cell cycle arrest through the inhibition of CDK4 and CDK6 expression and increasing apoptosis. It also decreased the expression of the PI3K/AKT/mTOR pathway and the NF-κβ/TGF-β pathway, inhibited CK18 and COX2 activity, increased the concentration of BAX and P53 proteins, and decreased Bcl-2, IL-6, IL-1β, MMP2, and MMP9. These findings suggested that CDK4/6i abemaciclib and radiotherapy may have an impact on breast cancer cell lines.

## Supplementary Information

Below is the link to the electronic supplementary material.

Supplementary file1 (HTM 9 KB)

Supplementary file2 (HTM 9 KB)

Supplementary file3 (HTM 9 KB)

Supplementary file4 (HTM 9 KB)

Supplementary file5 (HTM 9 KB)

Supplementary file6 (HTM 9 KB)

Supplementary file7 (HTM 9 KB)

## Data Availability

All source data for this work (or generated in this study) are available upon reasonable request.

## References

[CR1] Aggarwal S, Verma SS, Aggarwal S, Gupta SC (2021) Drug repurposing for breast cancer therapy: old weapon for new battle. Semin Cancer Biol 68:8–20 31550502 10.1016/j.semcancer.2019.09.012 PMC7128772

[CR2] Alian DME, Helmy MW, Haroun M, Moussa N (2024) Modulation of autophagy and apoptosis can contribute to the anticancer effect of abemaciclib/celecoxib combination in colon cancer cells. Med Oncol 41(2):43 38170401 10.1007/s12032-023-02288-z PMC10764487

[CR3] Anwer MK, Fatima F, Ahmed MM, Aldawsari MF, Alali AS, Kalam MA, Alshamsan A, Alkholief M, Malik A, Az A, Al-Shdefat R (2022) Abemaciclib-loaded ethylcellulose based nanosponges for sustained cytotoxicity against MCF-7 and MDA-MB-231 human breast cancer cells lines. Saudi Pharm J 30(6):726–734 35812154 10.1016/j.jsps.2022.03.019 PMC9257851

[CR4] Asghar U, Witkiewicz AK, Turner NC, Knudsen ES (2015) The history and future of targeting cyclin-dependent kinases in cancer therapy. Nat Rev Drug Discov 14(2):130–146 25633797 10.1038/nrd4504 PMC4480421

[CR5] Aubrey BJ, Kelly GL, Janic A, Herold MJ, Strasser A (2018) How does p53 induce apoptosis and how does this relate to p53-mediated tumour suppression? Cell Death Differ 25(1):104–113 29149101 10.1038/cdd.2017.169 PMC5729529

[CR6] Barkat HA, Das SS, Barkat MA, Beg S, Hadi HA (2020) Selective targeting of cancer signaling pathways with nanomedicines: challenges and progress. Future Oncol 16:2959–2979 32805124 10.2217/fon-2020-0198

[CR7] Barker HE, Paget JT, Khan AA, Harrington KJ (2015) The tumour microenvironment after radiotherapy: mechanisms of resistance and recurrence. Nat Rev Cancer 15(7):409–425 26105538 10.1038/nrc3958 PMC4896389

[CR8] Becherini C, Visani L, Caini S, Bhattacharya IS, Kirby AM, Marta GN, Morgan G, Salvestrini V, Coles CE, Cortes J, Curigliano G (2023) Safety profile of cyclin-dependent kinase (CDK) 4/6 inhibitors with concurrent radiation therapy: a systematic review and meta-analysis. Cancer Treat Rev 119:102586 37336117 10.1016/j.ctrv.2023.102586

[CR9] Bilgin B, Sendur MA, Şener Dede D, Akıncı MB, Yalçın B (2017) A current and comprehensive review of cyclin-dependent kinase inhibitors for the treatment of metastatic breast cancer. Curr Med Res Opin 33(9):1559–1569 28657360 10.1080/03007995.2017.1348344

[CR10] Blanc C, Deveraux QL, Krajewski S, Jänicke RU, Porter AG, Reed JC, Jaggi R, Marti A (2000) Caspase-3 is essential for procaspase-9 processing and cisplatin-induced apoptosis of MCF-7 breast cancer cells. Can Res 60(16):4386–4390 10969782

[CR11] Brentnall M, Rodriguez-Menocal L, De Guevara RL, Cepero E, Boise LH (2013) Caspase-9, caspase-3 and caspase-7 have distinct roles during intrinsic apoptosis. BMC Cell Biol 14:1–9 23834359 10.1186/1471-2121-14-32 PMC3710246

[CR12] Buege JA, Aust SD (1978) Microsomal lipid peroxidation. Methods Enzymol 52:302–310 672633 10.1016/s0076-6879(78)52032-6

[CR13] Carlos-Reyes A, Muñiz-Lino MA, Romero-Garcia S, López-Camarillo C, Hernández-de la Cruz ON (2021) Biological adaptations of tumor cells to radiation therapy. Front Oncol 11:718636 34900673 10.3389/fonc.2021.718636 PMC8652287

[CR14] Chen J (2016) The cell-cycle arrest and apoptotic functions of p53 in tumor initiation and progression. Cold Spring Harb Perspect Med 6:a026104 26931810 10.1101/cshperspect.a026104 PMC4772082

[CR15] Corona SP, Generali D (2018) Abemaciclib: a CDK4/6 inhibitor for the treatment of HR+/HER2- advanced breast cancer. Drug Desg Dev Therapy 12:321–330 10.2147/DDDT.S137783 PMC5818877 29497278

[CR16] Cretella D, Ravelli A, Fumarola C, La Monica S, Digiacomo G, Cavazzoni A, Alfieri R, Biondi A, Generali D, Bonelli M, Petronini PG (2018) The anti-tumor efficacy of CDK4/6 inhibition is enhanced by the combination with PI3K/AKT/mTOR inhibitors through impairment of glucose metabolism in TNBC cells. J Exp Clin Cancer Res 37:1–12 29587820 10.1186/s13046-018-0741-3 PMC5872523

[CR17] Deng W, Peng W, Wang T, Chen J, Zhu S (2019) Overexpression of MMPs functions as a prognostic biomarker for oral cancer patients: a systematic review and meta-analysis. Oral Health Prev Dent 17(6):505–514 31825023 10.3290/j.ohpd.a43636

[CR18] Devanaboyina M, Kaur J, Whiteley E, Lin L, Einloth K, Morand S, Stanbery L, Hamouda D, Nemunaitis J (2022) NF-κB signaling in tumor pathways focusing on breast and ovarian cancer. Oncol Rev 16:10568 36531159 10.3389/or.2022.10568 PMC9756851

[CR19] Ellman GL (1959) Tissue sulfhydryl groups. Arch Biochem Biophys 17:214–226 10.1016/0003-9861(59)90090-6 13650640

[CR20] Eskiler GG, Ozkan AD, Haciefendi A, Bilir C (2022) Mechanisms of abemaciclib, a CDK4/6 inhibitor, induced apoptotic cell death in prostate cancer cells in vitro. Transl Oncol 15(1):101243 34649150 10.1016/j.tranon.2021.101243 PMC8517924

[CR21] Franco R, Cao JQ, Yassa M, Hijal T (2023) Safety of CDK4/6 inhibitors combined with radiotherapy in patients with metastatic breast cancer: a review of the literature. Curr Oncol 30(6):5485–5496 37366898 10.3390/curroncol30060415 PMC10297034

[CR22] Galluzzi L, Kepp O, Kroemer G (2016) Mitochondrial regulation of cell death: a phylogenetically conserved control. Microb Cell 3:101–108 28357340 10.15698/mic2016.03.483 PMC5349020

[CR23] George MA, Qureshi S, Omene C, Toppmeyer DL, Ganesan S (2021) Clinical and pharmacologic differences of CDK4/6 inhibitors in breast cancer. Front Oncol 11:693104 34327137 10.3389/fonc.2021.693104 PMC8313476

[CR24] Ha SA, Lee YS, Kim HK, Yoo J, Kim S, Gong GH, Lee YK, Kim JW (2012) The prognostic potential of keratin 18 in breast cancer associated with tumor dedifferentiation, and the loss of estrogen and progesterone receptors. Cancer Biomark 10(5):219–231 10.3233/CBM-2012-0250 PMC13016247 22699783

[CR25] Halkett G, O’Connor M, Jefford M, Aranda S, Merchant S, Spry N, Kane R, Shaw T, Youens D, Moorin R, Schofield P (2018) RT Prepare: a radiation therapist-delivered intervention reduces psychological distress in women with breast cancer referred for radiotherapy. Br J Cancer 118(12):1549–1558 29855611 10.1038/s41416-018-0112-z PMC6008448

[CR26] Haussmann J, Corradini S, Nestle-Kraemling C, Bölke E, Njanang FJD, Tamaskovics B, Orth K, Ruckhaeberle E, Fehm T, Mohrmann S, Simiantonakis I (2020) Recent advances in radiotherapy of breast cancer. Radiat Oncol 15:1–10 10.1186/s13014-020-01501-x PMC7106718 32228654

[CR27] Hellinger JW, Schömel F, Buse JV, Lenz C, Bauerschmitz G, Emons G, Gründker C (2020) Identification of drivers of breast cancer invasion by secretome analysis: insight into CTGF signaling. Sci Rep 10(1):17889 33087801 10.1038/s41598-020-74838-8 PMC7578015

[CR28] Hino H, Iriyama N, Kokuba H, Kazama H, Moriya S, Takano N, Hiramoto M, Aizawa S, Miyazawa K (2020) Abemaciclib induces atypical cell death in cancer cells characterized by formation of cytoplasmic vacuoles derived from lysosomes. Cancer Sci 111(6):2132–2145 32304130 10.1111/cas.14419 PMC7293084

[CR29] Huang RX, Zhou PK (2020) DNA damage response signaling pathways and targets for radiotherapy sensitization in cancer. Signal Transduct Target Ther 5(1):60 32355263 10.1038/s41392-020-0150-x PMC7192953

[CR30] Jayanthan A, Yue L, Huynh MM, Los G, Dunn SE (2022) PMD-026, a first in class oral RSK inhibitor, demonstrates activity against hormone receptor positive breast cancer with acquired CDK4/6 inhibitor resistance. Cancer Res 82(12_Supplement):5378–5378

[CR31] Jiang H, Li H (2021) Prognostic values of tumoral MMP2 and MMP9 overexpression in breast cancer: a systematic review and meta-analysis. BMC Cancer 21:1–3 33568081 10.1186/s12885-021-07860-2 PMC7877076

[CR32] Jiao Y, Cao F, Liu H (2022) Radiation-induced cell death and its mechanisms. Health Phys 123(5):376–386 36069830 10.1097/HP.0000000000001601 PMC9512240

[CR33] Johnston SR, Toi M, O’Shaughnessy J, Rastogi P, Campone M, Neven P, Huang CS, Huober J, Jaliffe GG, Cicin I, Tolaney SM (2023) Abemaciclib plus endocrine therapy for hormone receptor-positive, HER2-negative, node-positive, high-risk early breast cancer (monarchE): results from a preplanned interim analysis of a randomised, open-label, phase 3 trial. Lancet Oncol 24(1):77–90 36493792 10.1016/S1470-2045(22)00694-5 PMC11200328

[CR34] Kawiak A, Kostecka A (2022) Regulation of Bcl-2 family proteins in estrogen receptor-positive breast cancer and their implications in endocrine therapy. Cancers 14(2):279 35053443 10.3390/cancers14020279 PMC8773933

[CR35] Kim BG, Malek E, Choi SH, Ignatz-Hoover JJ, Driscoll JJ (2021a) Novel therapies emerging in oncology to target the TGF-β pathway. J Hematol Oncol 14:1–20 33823905 10.1186/s13045-021-01053-x PMC8022551

[CR36] Kim KN, Shah P, Clark A, Freedman GM, Dastgheyb S, Barsky AR, Dreyfuss AD, Taunk NK (2021b) Safety of cyclin-dependent kinase4/6 inhibitor combined with palliative radiotherapy in patients with metastatic breast cancer. Breast 60:163–167 34653725 10.1016/j.breast.2021.10.001 PMC8527028

[CR37] Kulsoom B, Shamsi TS, Afsar NA, Memon Z, Ahmed N, Hasnain SN (2018) Bax, Bcl-2, and Bax/Bcl-2 as prognostic markers in acute myeloid leukemia: are we ready for Bcl-2-directed therapy? Cancer Manag Res. 10:403–416 29535553 10.2147/CMAR.S154608 PMC5841349

[CR38] Li J, Goh EL, He J, Li Y, Fan Z, Yu Z, Yuan P, Liu DX (2023) Emerging intrinsic therapeutic targets for metastatic breast cancer. Biology 12(5):697 37237509 10.3390/biology12050697 PMC10215321

[CR39] Li G, Guo J, Mou Y, Luo Q, Wang X, Xue W, Hou T, Zeng T, Yang Y (2024) Keratin gene signature expression drives epithelial-mesenchymal transition through enhanced TGF-β signaling pathway activation and correlates with adverse prognosis in lung adenocarcinoma. Heliyon. 10(3):e24549 38322947 10.1016/j.heliyon.2024.e24549 PMC10844058

[CR40] Lin F, Luo J, Gao W, Wu J, Shao Z, Wang Z, Meng J, Ou Z, Yang G (2013) COX-2 promotes breast cancer cell radioresistance via p38/MAPK-mediated cellular anti-apoptosis and invasiveness. Tumor Biol 34:2817–2826 10.1007/s13277-013-0840-x 23771849

[CR41] Liu Y, Zhao R, Fang S, Li Q, Jin Y, Liu B (2021) Abemaciclib sensitizes HPV-negative cervical cancer to chemotherapy via specifically suppressing CDK4/6-Rb-E2F and mTOR pathways. Fundam Clin Pharmacol 35(1):156–164 32446293 10.1111/fcp.12574

[CR42] Livak KJ, Schmittgen TD (2001) Analysis of relative gene expression data using real-time quantitative PCR and the 2− ΔΔCT method. Methods 25:402–408 11846609 10.1006/meth.2001.1262

[CR43] Mahmoud A, Casciati A, Bakar ZA, Hamzah H, Ahmad TAT, Noor MHM (2023) The detection of DNA damage response in MCF7 and MDA-MB-231 breast cancer cell lines after X-ray exposure. Genome Integr 14:e20220001 38025521 10.14293/genint.14.1.001 PMC10557035

[CR44] Man SM, Kanneganti TD (2016) Converging roles of caspases in inflammasome activation, cell death and innate immunity. Nat Rev Immunol 16(1):7–21 26655628 10.1038/nri.2015.7 PMC4915362

[CR45] Manore SG, Doheny DL, Wong GL, Lo HW (2022) IL-6/JAK/STAT3 signaling in breast cancer metastasis: biology and treatment. Front Oncol 12:866014 35371975 10.3389/fonc.2022.866014 PMC8964978

[CR46] Masoudi-Khoram N, Abdolmaleki P, Hosseinkhan N, Nikoofar A, Mowla SJ, Monfared H, Baldassarre G (2020) Differential miRNAs expression pattern of irradiated breast cancer cell lines is correlated with radiation sensitivity. Sci Rep 10(1):9054–9066 32493932 10.1038/s41598-020-65680-z PMC7270150

[CR47] Meattini I, Livi L, Lorito N, Becherini C, Bacci M, Visani L, Fozza A, Belgioia L, Loi M, Mangoni M, Lambertini M (2022) Integrating radiation therapy with targeted treatments for breast cancer: from bench to bedside. Cancer Treat Rev 108:102417 35623219 10.1016/j.ctrv.2022.102417

[CR48] Miranda KM, Espy MG, Wink DA (2001) A rapid, simple spectrophotometric method for simultaneous detection of nitrate and nitrite. Nitric Oxide Biol Chem 5:62–71 10.1006/niox.2000.0319 11178938

[CR49] Naz S, Sowers A, Choudhuri R, Wissler M, Gamson J, Mathias A, Cook JA, Mitchell JB (2018) Abemaciclib, a selective CDK4/6 inhibitor, enhances the radiosensitivity of non–small cell lung cancer in vitro and in vivo. Clin Cancer Res 24(16):3994–4005 29716919 10.1158/1078-0432.CCR-17-3575 PMC6137329

[CR50] Naz S, Cook JA, Mitchell JB (2019) Abemaciclib: a multi-functional radiation modifier. Oncotarget 10(12):1230–1232 30815224 10.18632/oncotarget.26652 PMC6383816

[CR51] Niu Y, Xu J, Sun T (2019) Cyclin-dependent kinases 4/6 inhibitors in breast cancer: current status, resistance, and combination strategies. J Cancer 10:5504 31632494 10.7150/jca.32628 PMC6775706

[CR52] O’Brien N, Conklin D, Beckmann R, Luo T, Chau K, Thomas J, Mc Nulty A, Marchal C, Kalous O, von Euw E, Hurvitz S (2018) Preclinical activity of abemaciclib alone or in combination with antimitotic and targeted therapies in breast cancer. Mol Cancer Ther 17(5):897–907 29483214 10.1158/1535-7163.MCT-17-0290

[CR53] Orning P, Lien E (2021) Multiple roles of caspase-8 in cell death, inflammation, and innate immunity. J Leukoc Biol 109:121–141 32531842 10.1002/JLB.3MR0420-305R PMC8664275

[CR54] Ozman Z, Guney Eskiler G, Sekeroglu MR (2021) In vitro therapeutic effects of abemaciclib on triple-negative breast cancer cells. J Biochem Mol Toxicol 35(9):e22858 34309953 10.1002/jbt.22858

[CR55] Pavitra E, Kancharla J, Gupta VK, Prasad K, Sung JY, Kim J, Tej MB, Choi R, Lee JH, Han YK, Raju GSR (2023) The role of NF-κB in breast cancer initiation, growth, metastasis, and resistance to chemotherapy. Biomed Pharmacother 163:114822 37146418 10.1016/j.biopha.2023.114822

[CR56] Pesch AM, Pierce LJ, Speers CW (2021) Modulating the radiation response for improved outcomes in breast cancer. JCO Precis Oncol 5:245–262 10.1200/PO.20.00297 PMC8232831 34250414

[CR57] Piezzo M, Cocco S, Caputo R, Cianniello D, Gioia GD, Lauro VD, Fusco G, Martinelli C, Nuzzo F, Pensabene M, Laurentiis MD (2020) Targeting cell cycle in breast cancer: CDK4/6 inhibitors. Int J Mol Sci 21(18):6479 32899866 10.3390/ijms21186479 PMC7554788

[CR58] Riley JS, Quarato G, Cloix C, Lopez J, O’Prey J, Pearson M, Chapman J, Sesaki H, Carlin LM, Passos JF, Wheeler AP (2018) Mitochondrial inner membrane permeabilisation enables mt DNA release during apoptosis. EMBO J 37(17):e99238 30049712 10.15252/embj.201899238 PMC6120664

[CR59] Saha T, Lukong KE (2022) Breast cancer stem-like cells in drug resistance: a review of mechanisms and novel therapeutic strategies to overcome drug resistance. Front Oncol 12:856974 35392236 10.3389/fonc.2022.856974 PMC8979779

[CR60] Samani RK, Tavakoli MB, Maghsoudinia F, Motaghi H, Hejazi SH, Mehrgardi MA (2020) Trastuzumab and folic acid functionalized gold nanoclusters as a dual-targeted radiosensitizer for megavoltage radiation therapy of human breast cancer. Eur J Pharm Sci 153:105487 32707173 10.1016/j.ejps.2020.105487

[CR61] Shi R, Liu L, Wang F, He Y, Niu Y, Wang C, Zhang X, Zhang X, Zhang H, Chen M, Wang Y (2020) Downregulation of cytokeratin 18 induces cellular partial EMT and stemness through increasing EpCAM expression in breast cancer. Cell Signal 76:109810 33069797 10.1016/j.cellsig.2020.109810

[CR62] Shikanai A, Horimoto Y, Ishizuka Y, Uomori T, Nakai K, Arakawa A, Saito M (2022) Clinicopathological features related to the efficacy of CDK4/6 inhibitor-based treatments in metastatic breast cancer. Breast Cancer Basic Clin Res 16:1–9 10.1177/11782234211065148 PMC8738870 35002243

[CR63] Skehan P, Storeng R, Scudiero D, Monks A, McMahon J, Vistica D, Warren JT, Bokesch H, Kenney S, Boyd MR (1990) New colorimetric cytotoxicity assay for anticancer-drug screening. JNCI J Natl Cancer Inst. 82(13):1107–1112 2359136 10.1093/jnci/82.13.1107

[CR64] Torres-Guzmán R, Calsina B, Hermoso A, Baquero C, Alvarez B, Amat J, McNulty AM, Gong X, Boehnke K, Du J, de Dios A (2017) Preclinical characterization of abemaciclib in hormone receptor positive breast cancer. Oncotarget 8(41):69493 29050219 10.18632/oncotarget.17778 PMC5642494

[CR65] Tsai CC, Wu SB, Kau HC, Wei YH (2018) Essential role of connective tissue growth factor (CTGF) in transforming growth factor-β1 (TGF-β1)-induced myofibroblast transdifferentiation from Graves’ orbital fibroblasts. Sci Rep 8(1):7276 29739987 10.1038/s41598-018-25370-3 PMC5940888

[CR66] Wagner V, Gil J (2020) Senescence as a therapeutically relevant response to CDK4/6 inhibitors. Oncogene 39:5165–5176 32541838 10.1038/s41388-020-1354-9 PMC7610384

[CR67] Wu X, Sun L, Xu F (2023) NF-κB in cell deaths, therapeutic resistance and nanotherapy of tumors: recent advances. Pharmaceuticals 16(6):783 37375731 10.3390/ph16060783 PMC10302521

[CR68] Yang J, Gao S, Xu J, Zhu J (2018) Prognostic value and clinicopathological significance of serum-and tissue-based cytokeratin 18 express level in breast cancer: a meta-analysis. Biosci Rep 38(2):BSR20171145 29437899 10.1042/BSR20171145 PMC5861326

[CR69] Zhang L, Bailleul J, Yazal T, Dong K, Sung D, Dao A, Gosa L, Nathanson D, Bhat K, Duhachek-Muggy S, Alli C (2019) PK-M2-mediated metabolic changes in breast cancer cells induced by ionizing radiation. Breast Cancer Res Treat 178:75–86 31372790 10.1007/s10549-019-05376-9 PMC6790295

[CR70] Zhu J, Zhang X, Ai L, Yuan R, Ye J (2019) Clinicohistopathological implications of MMP/VEGF expression in retinoblastoma: a combined meta-analysis and bioinformatics analysis. J Transl Med 17:1–14 31311559 10.1186/s12967-019-1975-3 PMC6636009

[CR71] Ziegler E, Hansen MT, Haase M, Emons G, Gründker C (2014) Generation of MCF-7 cells with aggressive metastatic potential in vitro and in vivo. Breast Cancer Res Treat 148:269–277 25292421 10.1007/s10549-014-3159-4

