# Supplementary material for: Effect of palliative radiotherapy and cyclin-dependent kinase 4/6 inhibitor on breast cancer cell lines
Source: Naunyn Schmiedebergs Arch Pharmacol. 2025 Mar 4;398(8):10753–68. doi: 10.1007/s00210-025-03878-6 (PMC12350456; doi:10.1007/s00210-025-03878-6)
Supplement: Supplementary file 1 — Supplementary file1 (HTM 9 KB) [file 210_2025_3878_MOESM1_ESM.htm]

CompuSyn Report


CompuSyn Report

|  |  |
| --- | --- |
| Experiment Name: | MDA-MB-231 |
| Date: | 10/1/2025 |
| File Name: | D:\Work\DR HEBA RAD\MDA-MB-231.cse |
| Description | Combination between Abemaciclib and 2.4.6.10 GY |

|  |  |
| --- | --- |
| Drug: | Abemaciclib (Abe) [�M] |
| Drug: | GY (GY) [Gy] |
| Drug Combo: | Combination index (Comb) (Abe+GY) |

---

Data for Drug: Abe [�M]

| Dose | Effect |
| --- | --- |
| 1.56 | 0.19 |
| 3.12 | 0.22 |
| 6.25 | 0.226 |
| 12.5 | 0.297 |
| 25.0 | 0.505 |
| 50.0 | 0.719 |

6 data points entered.

|  |  |
| --- | --- |
| X-int: | 1.36430 |
| Y-int: | -0.9090 +/- 0.14851 |
| m: | 0.66626 +/- 0.13790 |
| Dm: | 23.1367 |
| r: | 0.92397 |

---

Data for Drug: GY [Gy]

| Dose | Effect |
| --- | --- |
| 2.0 | 0.02 |
| 6.0 | 0.14 |
| 10.0 | 0.206 |

3 data points entered.

|  |  |
| --- | --- |
| X-int: | 1.32006 |
| Y-int: | -2.1506 +/- 0.17609 |
| m: | 1.62919 +/- 0.23419 |
| Dm: | 20.8959 |
| r: | 0.98983 |

---

Data for Non-Constant Combo: Comb (Abe+GY)

| Dose Abe | Dose GY | Effect |
| --- | --- | --- |
| 1.56 | 2.0 | 0.4 |
| 3.12 | 2.0 | 0.42 |
| 6.25 | 2.0 | 0.4 |
| 12.5 | 2.0 | 0.44 |
| 25.0 | 2.0 | 0.8 |
| 50.0 | 2.0 | 0.82 |

6 data points entered.

---

Dose-Effect Curve  


---

Median-Effect Plot  


---

CI Data for Non-Constant Combo: Comb (Abe+GY)

| Dose Abe | Dose GY | Effect | CI |
| --- | --- | --- | --- |
| 1.56 | 2.0 | 0.4 | 0.24667 |
| 3.12 | 2.0 | 0.42 | 0.33559 |
| 6.25 | 2.0 | 0.4 | 0.61921 |
| 12.5 | 2.0 | 0.44 | 0.88689 |
| 25.0 | 2.0 | 0.8 | 0.17577 |
| 50.0 | 2.0 | 0.82 | 0.25968 |

---

Combination Index Plot  


---

DRI Data for Non-Constant Combo: Comb (Abe+GY)

| Fa | Dose Abe | Dose GY | DRI Abe | DRI GY |
| --- | --- | --- | --- | --- |
| 0.4 | 12.5893 | 16.2921 | 8.07007 | 8.14604 |
| 0.42 | 14.2529 | 17.1404 | 4.56824 | 8.57018 |
| 0.4 | 12.5893 | 16.2921 | 2.01429 | 8.14604 |
| 0.44 | 16.1102 | 18.0208 | 1.28882 | 9.01042 |
| 0.8 | 185.329 | 48.9335 | 7.41316 | 24.4667 |
| 0.82 | 225.277 | 52.9998 | 4.50554 | 26.4999 |

---

DRI Plot for Non-Constant Combo: Comb (Abe+GY)  


---

Normalized Isobologram for Combo: Comb (Abe+GY)  


---

Summary Table

|  |  |
| --- | --- |
| Experiment Name: | MDA-MB-231 |
| Date: | 10/1/2025 |
| File Name: | D:\Work\DR HEBA RAD\MDA-MB-231.cse |
| Description | Combination between Abemaciclib and 2.4.6.10 GY |

|  |  |
| --- | --- |
| Drug: | Abemaciclib (Abe) [�M] |
| Drug: | GY (GY) [Gy] |
| Drug Combo: | Combination index (Comb) (Abe+GY) |

---

| Drug/Combo | Dm | m | r |
| --- | --- | --- | --- |
| Abe | 23.1367 | 0.66626 | 0.92397 |
| GY | 20.8959 | 1.62919 | 0.98983 |

---

|  |  |  |  |  |
| --- | --- | --- | --- | --- |
|  | CI values at: | | | |
| Combo | ED50 | ED75 | ED90 | ED95 |

---

Data for Fa = 0.5

| Drug/Combo | CI value | Dose Abe | Dose GY |
| --- | --- | --- | --- |
| Abe |  | 23.1367 |
| GY |  |  | 20.8959 |

---

Data for Fa = 0.75

| Drug/Combo | CI value | Dose Abe | Dose GY |
| --- | --- | --- | --- |
| Abe |  | 120.343 |
| GY |  |  | 41.0127 |

---

Data for Fa = 0.9

| Drug/Combo | CI value | Dose Abe | Dose GY |
| --- | --- | --- | --- |
| Abe |  | 625.951 |
| GY |  |  | 80.4963 |

---

Data for Fa = 0.95

| Drug/Combo | CI value | Dose Abe | Dose GY |
| --- | --- | --- | --- |
| Abe |  | 1921.34 |
| GY |  |  | 127.339 |

---

Data for Fa = 0.97

| Drug/Combo | CI value | Dose Abe | Dose GY |
| --- | --- | --- | --- |
| Abe |  | 4267.39 |
| GY |  |  | 176.476 |
