# Supplementary material for: Effect of palliative radiotherapy and cyclin-dependent kinase 4/6 inhibitor on breast cancer cell lines
Source: Naunyn Schmiedebergs Arch Pharmacol. 2025 Mar 4;398(8):10753–68. doi: 10.1007/s00210-025-03878-6 (PMC12350456; doi:10.1007/s00210-025-03878-6)
Supplement: Supplementary file 4 — Supplementary file4 (HTM 9 KB) [file 210_2025_3878_MOESM4_ESM.htm]

CompuSyn Report


CompuSyn Report

|  |  |
| --- | --- |
| Experiment Name: | MCF7 Combination |
| Date: | 10.1.2025 |
| File Name: | D:\Work\DR HEBA RAD\combination MCF7.cse |
| Description | MCF7 Abe Cobination 2, 6, 10 Gy |

|  |  |
| --- | --- |
| Drug: | Abemaciclib (Abe) [uM] |
| Drug: | GY (Gy) [Gy] |
| Drug Combo: | Abe+6GY (AbeGy) (Abe+Gy) |

---

Data for Drug: Abe [uM]

| Dose | Effect |
| --- | --- |
| 1.56 | 0.27 |
| 3.12 | 0.32 |
| 6.25 | 0.36 |
| 12.5 | 0.4 |
| 25.0 | 0.42 |
| 50.0 | 0.46 |

6 data points entered.

|  |  |
| --- | --- |
| X-int: | 1.94784 |
| Y-int: | -0.4521 +/- 0.01800 |
| m: | 0.23213 +/- 0.01671 |
| Dm: | 88.6821 |
| r: | 0.98979 |

---

Data for Drug: Gy [Gy]

| Dose | Effect |
| --- | --- |
| 2.0 | 0.01 |
| 6.0 | 0.073 |
| 10.0 | 0.108 |

3 data points entered.

|  |  |
| --- | --- |
| X-int: | 1.53235 |
| Y-int: | -2.4443 +/- 0.18500 |
| m: | 1.59514 +/- 0.24604 |
| Dm: | 34.0679 |
| r: | 0.98831 |

---

Data for Non-Constant Combo: AbeGy (Abe+Gy)

| Dose Abe | Dose Gy | Effect |
| --- | --- | --- |
| 1.56 | 6.0 | 0.39 |
| 3.12 | 6.0 | 0.44 |
| 6.25 | 6.0 | 0.47 |
| 12.5 | 6.0 | 0.54 |
| 25.0 | 6.0 | 0.55 |
| 50.0 | 6.0 | 0.58 |

6 data points entered.

---

Dose-Effect Curve  


---

Median-Effect Plot  


---

CI Data for Non-Constant Combo: AbeGy (Abe+Gy)

| Dose Abe | Dose Gy | Effect | CI |
| --- | --- | --- | --- |
| 1.56 | 6.0 | 0.39 | 0.35396 |
| 3.12 | 6.0 | 0.44 | 0.30429 |
| 6.25 | 6.0 | 0.47 | 0.30815 |
| 12.5 | 6.0 | 0.54 | 0.22992 |
| 25.0 | 6.0 | 0.55 | 0.27406 |
| 50.0 | 6.0 | 0.58 | 0.28422 |

---

Combination Index Plot  


---

DRI Data for Non-Constant Combo: AbeGy (Abe+Gy)

| Fa | Dose Abe | Dose Gy | DRI Abe | DRI Gy |
| --- | --- | --- | --- | --- |
| 0.39 | 12.9107 | 25.7371 | 8.27609 | 4.28952 |
| 0.44 | 31.3791 | 29.2878 | 10.0574 | 4.88130 |
| 0.47 | 52.8513 | 31.5962 | 8.45620 | 5.26603 |
| 0.54 | 176.939 | 37.6704 | 14.1552 | 6.27840 |
| 0.55 | 210.511 | 38.6349 | 8.42045 | 6.43915 |
| 0.58 | 356.221 | 41.7084 | 7.12442 | 6.95141 |

---

DRI Plot for Non-Constant Combo: AbeGy (Abe+Gy)  


---

Normalized Isobologram for Combo: AbeGy (Abe+Gy)  


---

Summary Table

|  |  |
| --- | --- |
| Experiment Name: | MCF7 Combination |
| Date: | 10.1.2025 |
| File Name: | D:\Work\DR HEBA RAD\combination MCF7.cse |
| Description | MCF7 Abe Cobination 2, 6, 10 Gy |

|  |  |
| --- | --- |
| Drug: | Abemaciclib (Abe) [uM] |
| Drug: | GY (Gy) [Gy] |
| Drug Combo: | Abe+6GY (AbeGy) (Abe+Gy) |

---

| Drug/Combo | Dm | m | r |
| --- | --- | --- | --- |
| Abe | 88.6821 | 0.23213 | 0.98979 |
| Gy | 34.0679 | 1.59514 | 0.98831 |

---

|  |  |  |  |  |
| --- | --- | --- | --- | --- |
|  | CI values at: | | | |
| Combo | ED50 | ED75 | ED90 | ED95 |

---

Data for Fa = 0.5

| Drug/Combo | CI value | Dose Abe | Dose Gy |
| --- | --- | --- | --- |
| Abe |  | 88.6821 |
| Gy |  |  | 34.0679 |

---

Data for Fa = 0.75

| Drug/Combo | CI value | Dose Abe | Dose Gy |
| --- | --- | --- | --- |
| Abe |  | 10075.2 |
| Gy |  |  | 67.8351 |

---

Data for Fa = 0.9

| Drug/Combo | CI value | Dose Abe | Dose Gy |
| --- | --- | --- | --- |
| Abe |  | 1144647 |
| Gy |  |  | 135.071 |

---

Data for Fa = 0.95

| Drug/Combo | CI value | Dose Abe | Dose Gy |
| --- | --- | --- | --- |
| Abe |  | 2.862E7 |
| Gy |  |  | 215.775 |

---

Data for Fa = 0.97

| Drug/Combo | CI value | Dose Abe | Dose Gy |
| --- | --- | --- | --- |
| Abe |  | 2.827E8 |
| Gy |  |  | 301.128 |
