# Supplementary material for: Effect of palliative radiotherapy and cyclin-dependent kinase 4/6 inhibitor on breast cancer cell lines
Source: Naunyn Schmiedebergs Arch Pharmacol. 2025 Mar 4;398(8):10753–68. doi: 10.1007/s00210-025-03878-6 (PMC12350456; doi:10.1007/s00210-025-03878-6)
Supplement: Supplementary file 5 — Supplementary file5 (HTM 9 KB) [file 210_2025_3878_MOESM5_ESM.htm]

CompuSyn Report


CompuSyn Report

|  |  |
| --- | --- |
| Experiment Name: | MCF7 Combination |
| Date: | 10.1.2025 |
| File Name: | D:\Work\DR HEBA RAD\combination MCF7.cse |
| Description | MCF7 Abe Cobination 2, 6, 10 Gy |

|  |  |
| --- | --- |
| Drug: | Abemaciclib (Abe) [uM] |
| Drug: | GY (Gy) [Gy] |
| Drug Combo: | Abe+2 Gy (Abe2gy) (Abe+Gy) |

---

Data for Drug: Abe [uM]

| Dose | Effect |
| --- | --- |
| 1.56 | 0.27 |
| 3.12 | 0.32 |
| 6.25 | 0.36 |
| 12.5 | 0.4 |
| 25.0 | 0.42 |
| 50.0 | 0.46 |

6 data points entered.

|  |  |
| --- | --- |
| X-int: | 1.94784 |
| Y-int: | -0.4521 +/- 0.01800 |
| m: | 0.23213 +/- 0.01671 |
| Dm: | 88.6821 |
| r: | 0.98979 |

---

Data for Drug: Gy [Gy]

| Dose | Effect |
| --- | --- |
| 2.0 | 0.01 |
| 6.0 | 0.073 |
| 10.0 | 0.108 |

3 data points entered.

|  |  |
| --- | --- |
| X-int: | 1.53235 |
| Y-int: | -2.4443 +/- 0.18500 |
| m: | 1.59514 +/- 0.24604 |
| Dm: | 34.0679 |
| r: | 0.98831 |

---

Data for Non-Constant Combo: Abe2gy (Abe+Gy)

| Dose Abe | Dose Gy | Effect |
| --- | --- | --- |
| 1.56 | 2.0 | 0.32 |
| 3.12 | 2.0 | 0.38 |
| 6.25 | 2.0 | 0.396 |
| 12.5 | 2.0 | 0.4 |
| 25.0 | 2.0 | 0.48 |
| 50.0 | 2.0 | 0.51 |

6 data points entered.

---

Dose-Effect Curve  


---

Median-Effect Plot  


---

CI Data for Non-Constant Combo: Abe2gy (Abe+Gy)

| Dose Abe | Dose Gy | Effect | CI |
| --- | --- | --- | --- |
| 1.56 | 2.0 | 0.32 | 0.54658 |
| 3.12 | 2.0 | 0.38 | 0.36968 |
| 6.25 | 2.0 | 0.396 | 0.51088 |
| 12.5 | 2.0 | 0.4 | 0.88417 |
| 25.0 | 2.0 | 0.48 | 0.45970 |
| 50.0 | 2.0 | 0.51 | 0.53181 |

---

Combination Index Plot  


---

DRI Data for Non-Constant Combo: Abe2gy (Abe+Gy)

| Fa | Dose Abe | Dose Gy | DRI Abe | DRI Gy |
| --- | --- | --- | --- | --- |
| 0.32 | 3.44816 | 21.2384 | 2.21036 | 10.6192 |
| 0.38 | 10.7629 | 25.0646 | 3.44965 | 12.5323 |
| 0.396 | 14.3882 | 26.1461 | 2.30212 | 13.0731 |
| 0.4 | 15.4612 | 26.4212 | 1.23689 | 13.2106 |
| 0.48 | 62.8177 | 32.4006 | 2.51271 | 16.2003 |
| 0.51 | 105.362 | 34.9331 | 2.10723 | 17.4666 |

---

DRI Plot for Non-Constant Combo: Abe2gy (Abe+Gy)  


---

Normalized Isobologram for Combo: Abe2gy (Abe+Gy)  


---

Summary Table

|  |  |
| --- | --- |
| Experiment Name: | MCF7 Combination |
| Date: | 10.1.2025 |
| File Name: | D:\Work\DR HEBA RAD\combination MCF7.cse |
| Description | MCF7 Abe Cobination 2, 6, 10 Gy |

|  |  |
| --- | --- |
| Drug: | Abemaciclib (Abe) [uM] |
| Drug: | GY (Gy) [Gy] |
| Drug Combo: | Abe+2 Gy (Abe2gy) (Abe+Gy) |

---

| Drug/Combo | Dm | m | r |
| --- | --- | --- | --- |
| Abe | 88.6821 | 0.23213 | 0.98979 |
| Gy | 34.0679 | 1.59514 | 0.98831 |

---

|  |  |  |  |  |
| --- | --- | --- | --- | --- |
|  | CI values at: | | | |
| Combo | ED50 | ED75 | ED90 | ED95 |

---

Data for Fa = 0.5

| Drug/Combo | CI value | Dose Abe | Dose Gy |
| --- | --- | --- | --- |
| Abe |  | 88.6821 |
| Gy |  |  | 34.0679 |

---

Data for Fa = 0.75

| Drug/Combo | CI value | Dose Abe | Dose Gy |
| --- | --- | --- | --- |
| Abe |  | 10075.2 |
| Gy |  |  | 67.8351 |

---

Data for Fa = 0.9

| Drug/Combo | CI value | Dose Abe | Dose Gy |
| --- | --- | --- | --- |
| Abe |  | 1144647 |
| Gy |  |  | 135.071 |

---

Data for Fa = 0.95

| Drug/Combo | CI value | Dose Abe | Dose Gy |
| --- | --- | --- | --- |
| Abe |  | 2.862E7 |
| Gy |  |  | 215.775 |

---

Data for Fa = 0.97

| Drug/Combo | CI value | Dose Abe | Dose Gy |
| --- | --- | --- | --- |
| Abe |  | 2.827E8 |
| Gy |  |  | 301.128 |
