# Supplementary material for: Effect of palliative radiotherapy and cyclin-dependent kinase 4/6 inhibitor on breast cancer cell lines
Source: Naunyn Schmiedebergs Arch Pharmacol. 2025 Mar 4;398(8):10753–68. doi: 10.1007/s00210-025-03878-6 (PMC12350456; doi:10.1007/s00210-025-03878-6)
Supplement: Supplementary file 6 — Supplementary file6 (HTM 9 KB) [file 210_2025_3878_MOESM6_ESM.htm]

CompuSyn Report


CompuSyn Report

|  |  |
| --- | --- |
| Experiment Name: | MDA-MB-231 |
| Date: | 10/1/2025 |
| File Name: | D:\Work\DR HEBA RAD\MDA-MB-231.cse |
| Description | Combination between Abemaciclib and 2.4.6.10 GY |

|  |  |
| --- | --- |
| Drug: | Abemaciclib (Abe) [�M] |
| Drug: | GY (GY) [Gy] |
| Drug Combo: | Combination 10 GY (COMB10) (Abe+GY) |

---

Data for Drug: Abe [�M]

| Dose | Effect |
| --- | --- |
| 1.56 | 0.19 |
| 3.12 | 0.22 |
| 6.25 | 0.226 |
| 12.5 | 0.297 |
| 25.0 | 0.505 |
| 50.0 | 0.719 |

6 data points entered.

|  |  |
| --- | --- |
| X-int: | 1.36430 |
| Y-int: | -0.9090 +/- 0.14851 |
| m: | 0.66626 +/- 0.13790 |
| Dm: | 23.1367 |
| r: | 0.92397 |

---

Data for Drug: GY [Gy]

| Dose | Effect |
| --- | --- |
| 2.0 | 0.02 |
| 6.0 | 0.14 |
| 10.0 | 0.206 |

3 data points entered.

|  |  |
| --- | --- |
| X-int: | 1.32006 |
| Y-int: | -2.1506 +/- 0.17609 |
| m: | 1.62919 +/- 0.23419 |
| Dm: | 20.8959 |
| r: | 0.98983 |

---

Data for Non-Constant Combo: COMB10 (Abe+GY)

| Dose Abe | Dose GY | Effect |
| --- | --- | --- |
| 1.56 | 10.0 | 0.48 |
| 3.12 | 10.0 | 0.49 |
| 6.25 | 10.0 | 0.55 |
| 12.5 | 10.0 | 0.56 |
| 25.0 | 10.0 | 0.7 |
| 50.0 | 10.0 | 0.8 |

6 data points entered.

---

Dose-Effect Curve  


---

Median-Effect Plot  


---

CI Data for Non-Constant Combo: COMB10 (Abe+GY)

| Dose Abe | Dose GY | Effect | CI |
| --- | --- | --- | --- |
| 1.56 | 10.0 | 0.48 | 0.57869 |
| 3.12 | 10.0 | 0.49 | 0.63366 |
| 6.25 | 10.0 | 0.55 | 0.62298 |
| 12.5 | 10.0 | 0.56 | 0.78891 |
| 25.0 | 10.0 | 0.7 | 0.58742 |
| 50.0 | 10.0 | 0.8 | 0.47415 |

---

Combination Index Plot  


---

DRI Data for Non-Constant Combo: COMB10 (Abe+GY)

| Fa | Dose Abe | Dose GY | DRI Abe | DRI GY |
| --- | --- | --- | --- | --- |
| 0.48 | 20.5175 | 19.8941 | 13.1523 | 1.98941 |
| 0.49 | 21.7883 | 20.3891 | 6.98343 | 2.03891 |
| 0.55 | 31.2684 | 23.6349 | 5.00295 | 2.36349 |
| 0.56 | 33.2277 | 24.2297 | 2.65821 | 2.42297 |
| 0.7 | 82.5284 | 35.1500 | 3.30114 | 3.51500 |
| 0.8 | 185.329 | 48.9335 | 3.70658 | 4.89335 |

---

DRI Plot for Non-Constant Combo: COMB10 (Abe+GY)  


---

Normalized Isobologram for Combo: COMB10 (Abe+GY)  


---

Summary Table

|  |  |
| --- | --- |
| Experiment Name: | MDA-MB-231 |
| Date: | 10/1/2025 |
| File Name: | D:\Work\DR HEBA RAD\MDA-MB-231.cse |
| Description | Combination between Abemaciclib and 2.4.6.10 GY |

|  |  |
| --- | --- |
| Drug: | Abemaciclib (Abe) [�M] |
| Drug: | GY (GY) [Gy] |
| Drug Combo: | Combination 10 GY (COMB10) (Abe+GY) |

---

| Drug/Combo | Dm | m | r |
| --- | --- | --- | --- |
| Abe | 23.1367 | 0.66626 | 0.92397 |
| GY | 20.8959 | 1.62919 | 0.98983 |

---

|  |  |  |  |  |
| --- | --- | --- | --- | --- |
|  | CI values at: | | | |
| Combo | ED50 | ED75 | ED90 | ED95 |

---

Data for Fa = 0.5

| Drug/Combo | CI value | Dose Abe | Dose GY |
| --- | --- | --- | --- |
| Abe |  | 23.1367 |
| GY |  |  | 20.8959 |

---

Data for Fa = 0.75

| Drug/Combo | CI value | Dose Abe | Dose GY |
| --- | --- | --- | --- |
| Abe |  | 120.343 |
| GY |  |  | 41.0127 |

---

Data for Fa = 0.9

| Drug/Combo | CI value | Dose Abe | Dose GY |
| --- | --- | --- | --- |
| Abe |  | 625.951 |
| GY |  |  | 80.4963 |

---

Data for Fa = 0.95

| Drug/Combo | CI value | Dose Abe | Dose GY |
| --- | --- | --- | --- |
| Abe |  | 1921.34 |
| GY |  |  | 127.339 |

---

Data for Fa = 0.97

| Drug/Combo | CI value | Dose Abe | Dose GY |
| --- | --- | --- | --- |
| Abe |  | 4267.39 |
| GY |  |  | 176.476 |
