# Supplementary material for: Effect of palliative radiotherapy and cyclin-dependent kinase 4/6 inhibitor on breast cancer cell lines
Source: Naunyn Schmiedebergs Arch Pharmacol. 2025 Mar 4;398(8):10753–68. doi: 10.1007/s00210-025-03878-6 (PMC12350456; doi:10.1007/s00210-025-03878-6)
Supplement: Supplementary file 7 — Supplementary file7 (HTM 9 KB) [file 210_2025_3878_MOESM7_ESM.htm]

CompuSyn Report


CompuSyn Report

|  |  |
| --- | --- |
| Experiment Name: | MCF7 Combination |
| Date: | 10.1.2025 |
| File Name: | D:\Work\DR HEBA RAD\combination MCF7.cse |
| Description | MCF7 Abe Cobination 2, 6, 10 Gy |

|  |  |
| --- | --- |
| Drug: | Abemaciclib (Abe) [uM] |
| Drug: | GY (Gy) [Gy] |
| Drug Combo: | Abe+10 (Abe10G) (Abe+Gy) |

---

Data for Drug: Abe [uM]

| Dose | Effect |
| --- | --- |
| 1.56 | 0.27 |
| 3.12 | 0.32 |
| 6.25 | 0.36 |
| 12.5 | 0.4 |
| 25.0 | 0.42 |
| 50.0 | 0.46 |

6 data points entered.

|  |  |
| --- | --- |
| X-int: | 1.94784 |
| Y-int: | -0.4521 +/- 0.01800 |
| m: | 0.23213 +/- 0.01671 |
| Dm: | 88.6821 |
| r: | 0.98979 |

---

Data for Drug: Gy [Gy]

| Dose | Effect |
| --- | --- |
| 2.0 | 0.01 |
| 6.0 | 0.073 |
| 10.0 | 0.108 |

3 data points entered.

|  |  |
| --- | --- |
| X-int: | 1.53235 |
| Y-int: | -2.4443 +/- 0.18500 |
| m: | 1.59514 +/- 0.24604 |
| Dm: | 34.0679 |
| r: | 0.98831 |

---

Data for Non-Constant Combo: Abe10G (Abe+Gy)

| Dose Abe | Dose Gy | Effect |
| --- | --- | --- |
| 1.56 | 10.0 | 0.4 |
| 3.12 | 10.0 | 0.46 |
| 6.25 | 10.0 | 0.52 |
| 12.5 | 10.0 | 0.54 |
| 25.0 | 10.0 | 0.56 |
| 50.0 | 10.0 | 0.61 |

6 data points entered.

---

Dose-Effect Curve  


---

Median-Effect Plot  


---

CI Data for Non-Constant Combo: Abe10G (Abe+Gy)

| Dose Abe | Dose Gy | Effect | CI |
| --- | --- | --- | --- |
| 1.56 | 10.0 | 0.4 | 0.47938 |
| 3.12 | 10.0 | 0.46 | 0.39477 |
| 6.25 | 10.0 | 0.52 | 0.32909 |
| 12.5 | 10.0 | 0.54 | 0.33611 |
| 25.0 | 10.0 | 0.56 | 0.35209 |
| 50.0 | 10.0 | 0.61 | 0.30383 |

---

Combination Index Plot  


---

DRI Data for Non-Constant Combo: Abe10G (Abe+Gy)

| Fa | Dose Abe | Dose Gy | DRI Abe | DRI Gy |
| --- | --- | --- | --- | --- |
| 0.4 | 15.4612 | 26.4212 | 9.91101 | 2.64212 |
| 0.46 | 44.4475 | 30.8099 | 14.2460 | 3.08099 |
| 0.52 | 125.196 | 35.8210 | 20.0313 | 3.58210 |
| 0.54 | 176.939 | 37.6704 | 14.1552 | 3.76704 |
| 0.56 | 250.629 | 39.6282 | 10.0252 | 3.96282 |
| 0.61 | 609.147 | 45.0953 | 12.1829 | 4.50953 |

---

DRI Plot for Non-Constant Combo: Abe10G (Abe+Gy)  


---

Normalized Isobologram for Combo: Abe10G (Abe+Gy)  


---

Summary Table

|  |  |
| --- | --- |
| Experiment Name: | MCF7 Combination |
| Date: | 10.1.2025 |
| File Name: | D:\Work\DR HEBA RAD\combination MCF7.cse |
| Description | MCF7 Abe Cobination 2, 6, 10 Gy |

|  |  |
| --- | --- |
| Drug: | Abemaciclib (Abe) [uM] |
| Drug: | GY (Gy) [Gy] |
| Drug Combo: | Abe+10 (Abe10G) (Abe+Gy) |

---

| Drug/Combo | Dm | m | r |
| --- | --- | --- | --- |
| Abe | 88.6821 | 0.23213 | 0.98979 |
| Gy | 34.0679 | 1.59514 | 0.98831 |

---

|  |  |  |  |  |
| --- | --- | --- | --- | --- |
|  | CI values at: | | | |
| Combo | ED50 | ED75 | ED90 | ED95 |

---

Data for Fa = 0.5

| Drug/Combo | CI value | Dose Abe | Dose Gy |
| --- | --- | --- | --- |
| Abe |  | 88.6821 |
| Gy |  |  | 34.0679 |

---

Data for Fa = 0.75

| Drug/Combo | CI value | Dose Abe | Dose Gy |
| --- | --- | --- | --- |
| Abe |  | 10075.2 |
| Gy |  |  | 67.8351 |

---

Data for Fa = 0.9

| Drug/Combo | CI value | Dose Abe | Dose Gy |
| --- | --- | --- | --- |
| Abe |  | 1144647 |
| Gy |  |  | 135.071 |

---

Data for Fa = 0.95

| Drug/Combo | CI value | Dose Abe | Dose Gy |
| --- | --- | --- | --- |
| Abe |  | 2.862E7 |
| Gy |  |  | 215.775 |

---

Data for Fa = 0.97

| Drug/Combo | CI value | Dose Abe | Dose Gy |
| --- | --- | --- | --- |
| Abe |  | 2.827E8 |
| Gy |  |  | 301.128 |
